# Basic leucine zipper gene *VvbZIP61* is expressed at a quantitative trait locus for high monoterpene content in grape berries

**DOI:** 10.1093/hr/uhad151

**Published:** 2023-07-18

**Authors:** Yuyu Zhang, Cuixia Liu, Xianju Liu, Zemin Wang, Yi Wang, Gan-yuan Zhong, Shaohua Li, Zhanwu Dai, Zhenchang Liang, Peige Fan

**Affiliations:** Beijing Key Laboratory of Grape Science and Enology, and CAS Key Laboratory of Plant Resources, Institute of Botany, Chinese Academy of Sciences, Beijing 100093, China; University of Chinese Academy of Sciences, Beijing 100049, China; Centre for Special Economic Plant Studies, Guangxi Institute of Botany, Guangxi Zhuang Autonomous Region and Chinese Academy of Sciences, Guilin 541006, Guangxi, China; Beijing Key Laboratory of Grape Science and Enology, and CAS Key Laboratory of Plant Resources, Institute of Botany, Chinese Academy of Sciences, Beijing 100093, China; University of Chinese Academy of Sciences, Beijing 100049, China; College of Life Science and Technology, Gansu Agricultural University, Lanzhou 730070, China; Beijing Key Laboratory of Grape Science and Enology, and CAS Key Laboratory of Plant Resources, Institute of Botany, Chinese Academy of Sciences, Beijing 100093, China; Grape Genetics Research Unit, USDA-ARS, Geneva 14456, USA; Beijing Key Laboratory of Grape Science and Enology, and CAS Key Laboratory of Plant Resources, Institute of Botany, Chinese Academy of Sciences, Beijing 100093, China; Beijing Key Laboratory of Grape Science and Enology, and CAS Key Laboratory of Plant Resources, Institute of Botany, Chinese Academy of Sciences, Beijing 100093, China; University of Chinese Academy of Sciences, Beijing 100049, China; Beijing Key Laboratory of Grape Science and Enology, and CAS Key Laboratory of Plant Resources, Institute of Botany, Chinese Academy of Sciences, Beijing 100093, China; University of Chinese Academy of Sciences, Beijing 100049, China; Beijing Key Laboratory of Grape Science and Enology, and CAS Key Laboratory of Plant Resources, Institute of Botany, Chinese Academy of Sciences, Beijing 100093, China; University of Chinese Academy of Sciences, Beijing 100049, China

## Abstract

The widely appreciated muscat flavor of grapes and wine is mainly attributable to the monoterpenes that accumulate in ripe grape berries. To identify quantitative trait loci (QTL) for grape berry monoterpene content, an F_1_ mapping population was constructed by a cross between two grapevine genotypes, one with neutral aroma berries (cv. ‘Beifeng’) and the other with a pronounced muscat aroma (elite *Vitis vinifera* line ‘3–34’). A high-density genetic linkage map spanning 1563.7 cM was constructed using 3332 SNP markers that were assigned to 19 linkage groups. Monoterpenes were extracted from the berry of the F_1_ progeny, then identified and quantified by gas chromatography–mass spectrometry. Twelve stable QTLs associated with the amounts of 11 monoterpenes in berries were thus identified. In parallel, the levels of RNA in berries from 34 diverse cultivars were estimated by RNA sequencing and compared to the monoterpene content of the berries. The expression of five genes mapping to stable QTLs correlated well with the monoterpene content of berries. These genes, including the basic leucine zipper *VvbZIP61* gene on chromosome 12, are therefore considered as potentially being involved in monoterpene metabolism. Overexpression of *VvbZIP61* in *Vitis amurensis* callus through Agrobacterium-mediated transformation significantly increased the accumulation of several monoterpenes in the callus, including nerol, linalool, geranial, geraniol, β-myrcene, and D-limonene. It is hypothesized that *VvbZIP61* expression acts to increase muscat flavor in grapes. These results advance our understanding of the genetic control of monoterpene biosynthesis in grapes and provide important information for the marker-assisted selection of aroma compounds in grape breeding.

## Introduction

Volatile compounds shape the aroma profiles of table grapes and wines and so are important quality characteristics [1, 2]. Among the different chemical families of volatile compounds [3, 4], monoterpenes are considered key to defining the attractive muscat aroma of some *Vitis vinifera* cultivars [5, 6]. The main monoterpenes in muscat-flavored grape cultivars include nerol, linalool, geraniol, citronellol, α-terpineol, and rose oxide [5, 7, 8]. Differences in monoterpene composition and content result in differences in aroma attributes [1, 5] and may be influenced by external factors such as the degree of exposure to sunlight [9, 10], variation in climatic conditions [11], the site of cultivation, and the methods employed in canopy management [12–14].

The genetic background of a grape cultivar is, however, the most critical factor determining both aroma content and composition, with the heritability of linalool, nerol, geraniol, and α-terpineol ranging from 72.3% to 98.9% [15]. Monoterpene content is typically controlled by multiple genes in grape berries and is expressed as a quantitative trait [16, 17]. A few studies have identified genomic loci linked with monoterpene levels in grapes. A major quantitative trait locus (QTL) for nerol, geraniol, and linalool on chromosome 5 [15], and minor QTLs associated with muscat aroma on several other chromosomes [15, 17–19] have been reported from previous studies. The QTL mapping of monoterpene variation in grapes was mostly based on information from random amplification of polymorphic DNA (RAPD) [20, 21], amplified restriction fragment polymorphism (AFLP) [21, 22], sequence characterized amplified region (SCAR) [23], and simple sequence repeats (SSR) markers [24–26], which all tend to have a low density of genome coverage and limiting the resolution and fine mapping of the causal polymorphisms [27–29]. Single nucleotide polymorphism (SNP) markers are a powerful system for constructing high-density genetic maps for QTL analysis [30], which could therefore be applied in grapes to explore the genetic determinism of monoterpene metabolism more comprehensively.

Genotyping by sequencing (GBS) is an approach for affordable access to obtain dense genome-wide SNP markers for large populations. It has been used for genetic studies in a large number of species [31–34]. For instance, grape using GBS have established high-density genetic maps, which significantly improved the precision and pertinence of QTL identification [35–41]. In addition, RNA sequencing (RNA-Seq) can provide precise and genome-wide quantification of transcript levels which can be used in combination with QTL analysis to efficiently explore candidate genes in the loci of interest [42–46].

**Figure 1:**
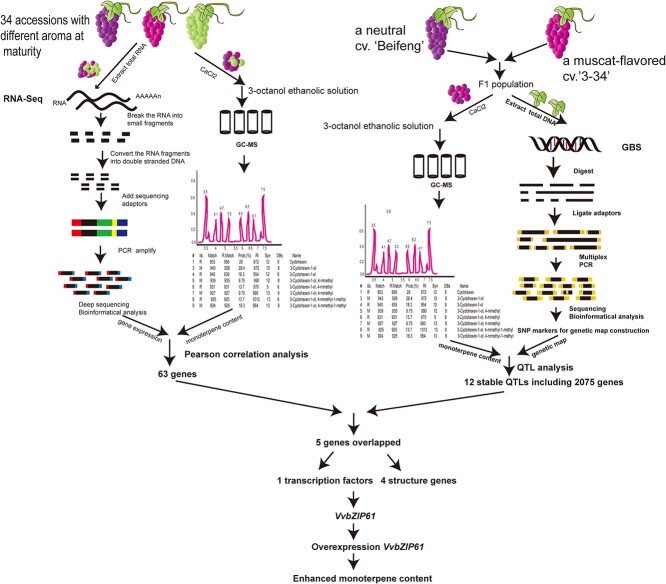
Workflow of this research.

In this study, we applied such an integrative approach to study the genetic regulation of monoterpenes in grape (Fig. 1). We focused on the inheritance of aroma traits from a cross between an elite line of *V. vinifera* noted for its strong muscat aroma and a neutral aroma cultivar (a hybrid between *Vitis thunbergii* and *V. vinifera*). The GBS approach was utilized to construct a genetic map of SNPs for the F_1_ segregating progeny. We quantified berry monoterpenes by gas chromatography–mass spectrometry (GC–MS) for each F_1_ genotype over 3 years to assess whether the QTLs were stable. In parallel, we sequenced the RNA in grapes from a range of cultivars and quantified the monoterpene content to correlate the expression of certain genes with monoterpene profiles. By integrating the QTL results and the phenotype-gene expression correlation analysis we obtained a common list of genes putatively involved in monoterpene accumulation traits (Fig. 1).

## Results

### Quantitative and qualitative variation in berry monoterpene content in an F_1_ grape population segregating for Muscat aroma components

Grape berries were harvested from 150 individual F_1_ progeny of a cross between *Vitis sp.* parents ‘Beifeng’ and ‘3–34’, cultivars that differ in their aroma characteristics. ‘Beifeng’ berries have a neutral aroma, while ‘3–34’ berries are noted for their strong muscat aroma. As expected, the GC–MS results showed that ‘Beifeng’ had low amounts of monoterpenes, while ‘3–34’ had various abundant monoterpenes characteristic of the muscat aroma. In the F_1_ population, a total of 17 monoterpenes were detected and quantified with GC–MS (Figs S1, S2, and S3, see online supplementary material). Among them, linalool was the highest amount, accounting for 17.1% of the total monoterpenes on average (Fig. S1, see online supplementary material). Rose oxide, nerol, and linalool 3,7-oxide accounted for 15.1%, 14.2%, and 10.2% of the total monoterpenes on average (Fig. S1, see online supplementary material). These four dominant components together accounted for well over half of the total monoterpenes. Amounts of 14 of the 17 monoterpenes showed continuous variation in the individuals of the F_1_ population over the three years (Fig. S2, see online supplementary material). The variation in each of these 14 monoterpenes was skewed slightly towards lower abundance, as determined by a statistical test of normal distribution, kurtosis, and skewness performed with the Shapiro–Wilk test [47] of R [48] (Fig. S2, see online supplementary material). To mitigate this skewness, square-root transformation, and log transformation were applied to the monoterpene content. However, it was found that even after these transformations, the majority of the values remained skewed. Consequently, we prefer to utilize the raw monoterpene content data for further analysis, acknowledging the presence of skewness in the dataset. The three remaining monoterpenes, namely nerol, linalool 3,7-oxide, and rose oxide were only detected in some of the F_1_ individuals (Figs S1 and S3, see online supplementary material). Interestingly, for these three monoterpenes, the ratios between the number of individuals with detectable levels to the number of individuals with non-detectable levels were 1:1 for nerol, 3:1 for linalool 3,7-oxide, and 3:1 for rose oxide, more characteristic of discrete variation (Fig. S3, Table S1, see online supplementary material). The Spearman correlation coefficients suggest that for F_1_ individuals, the contents of most monoterpene compounds were correlated with each other (Fig. S4, see online supplementary material).

### High-density genetic map construction in grape

**Figure 2:**
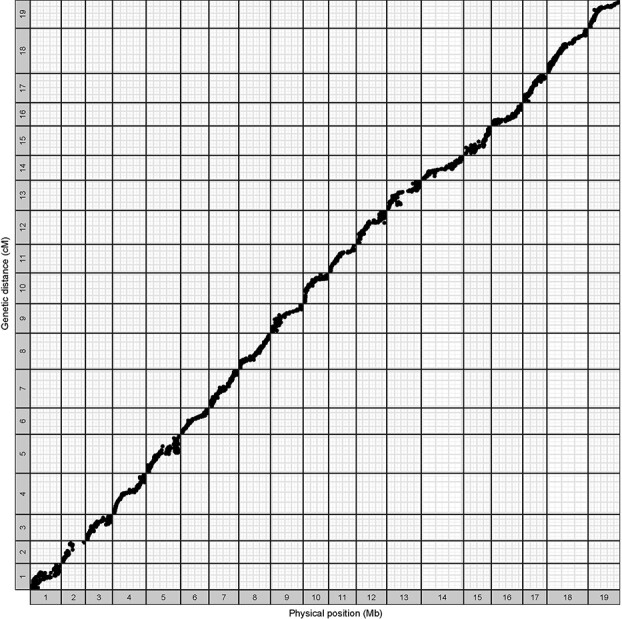
Relationship between given markers in genetic and physical maps of the 12X.v2 of the PN40024 reference genome. LG1 to LG19 represent the 19 genetic linkage groups; Chr1 to Chr19 represent the 19 physical maps of chromosomes.

To identify genome-wide SNPs from the grape cultivars used in the muscat aroma genetic study, the genomes of the 150 F_1_ individuals and their parents were digested with the restriction enzyme *ApeKI* and constructed 96-plex GBS libraries. The sequencing of the library produced 215 632 049 reads of 100 bp. The mean depth of coverage was 0.59, which was found to be lower compared to the depth of coverage in whole-genome sequencing (WGS). However, this data was enough for analysis. All the SNPs of 150 F_1_ progeny and parents were obtained using the TASSEL-GBS pipeline with the sequence data relative to the *V. vinifera* reference genome PN40024 12X.v2 [49]. A total of 80 512 SNPs were detected. A set of 10 372 high-quality SNPs, namely those with minor allele frequency (MAF) ≥0.05 and missing data ≤20% were used for linkage map construction with Joinmap 4.0 [50]. Markers with severely distorted loci (*P* ≤ 0.05) and those with 100% similarity were excluded. Nineteen strongly linked groups were identified using a threshold linkage logarithm of odds (LOD) score of 9.0. Finally, a total of 3332 SNPs were kept for genetic maps (Fig. S5, see online supplementary material). These markers were homozygous for one parent and heterozygous for another, or heterozygous for both parents (727 for lm × ll, 1582 for nn × np and 1023 for hk × hk, respectively). In the maternal map, 1717 markers were anchored on 19 linkage groups (LGs) spanning 1460 cM, with an average genetic distance of 0.9 cM. The paternal map consisted of 2741 SNPs distributed across 19 LGs spanning 1493.5 cM, with an average interval length of 0.5 cM. The integrated map was made by joining maternal and paternal maps using the Join Map version 4.0 [50]. The integrated map contained 19 LGs spanning 1563.7 cM with an average distance of 0.5 cM and 89% breadth of coverage of the reference genome (Table 1; Table S2, Fig. S6, see online supplementary material). The number of per linkage group SNPs ranged from 87 (LG2) to 251 (LG18) with an average of 175.4 (Table 1). The largest gaps were observed on LG2 (10.2 cM) and LG10 (9.32 cM) (Table S2, Fig. S6, see online supplementary material). The genetic and physical maps showed high correlation and good linearity (Fig. 2;Table S3, see online supplementary material).

**Table 1:** Properties of the genetic maps constructed for QTL detection in the F_1_ population of ‘Beifeng’ (maternal parent) and ‘3–34’ (paternal parent)

	Number of markers			Genetic distance (cM)			Avg. intermarker distance (cM)		
LGs	Beifeng	3–34	Integrated	Beifeng	3–34	Integrated	Beifeng	3–34	Integrated
LG1	181	191	194	59.0	54.4	69.8	0.3	0.3	0.4
LG2	59	79	87	46.0	25.8	60.2	0.8	0.3	0.7
LG3	100	155	217	59.0	71.1	69.8	0.6	0.5	0.3
LG4	44	185	202	47.1	109.4	108.9	1.1	0.6	0.5
LG5	94	111	196	96.5	90.3	103.3	1.0	0.8	0.5
LG6	64	182	173	107.2	80.8	70.4	1.7	0.4	0.4
LG7	117	157	245	108.7	95.9	102.3	0.9	0.6	0.4
LG8	100	148	212	97.2	69.0	95.5	1.0	0.5	0.5
LG9	53	133	121	85.4	97.1	78.2	1.6	0.7	0.6
LG10	77	113	146	82.1	73.4	82.1	1.0	0.7	0.6
LG11	55	86	128	72.3	70.7	75.4	1.3	0.8	0.6
LG12	67	117	163	73.1	94.2	89.7	1.1	0.8	0.6
LG13	112	190	181	84.6	87.5	80.1	0.8	0.5	0.4
LG14	101	128	167	71.7	63.6	65.6	0.7	0.5	0.4
LG15	89	112	149	75.9	70.5	78.6	0.9	0.6	0.5
LG16	92	119	158	62.1	56.8	61.6	0.7	0.5	0.4
LG17	67	164	183	72.0	77.4	77.3	1.1	0.5	0.4
LG18	176	229	251	105.1	117.3	120.3	0.6	0.5	0.5
LG19	69	142	159	55.0	88.3	74.6	0.8	0.6	0.5
Total	1717	2741	3332	1460	1493.5	1563.7	0.9	0.5	0.5

### QTL analysis

For the 14 individual monoterpenes and the total monoterpene content considered to be segregating as continuous traits, the significant LOD thresholds were estimated by 1000 permutations (Table S4, see online supplementary material). However, the results showed that only one QTL for nerol oxide passed the 1000 permutations LOD threshold. The significant LOD thresholds may appear due to the large skewness of the monoterpene content. As a consequence, none of the known QTLs for monoterpene content passed these thresholds (Table S5, see online supplementary material). One known QTL on LG12 for the linalool was found with LOD scores of 3.56 and 3.96, respectively (Table S5, see online supplementary material). To avoid excluding potential QTLs, based on the QTL detection power for the known QTLs the LOD of 3.5 was chosen as the final threshold for all further QTL identifications.

**Figure 3:**
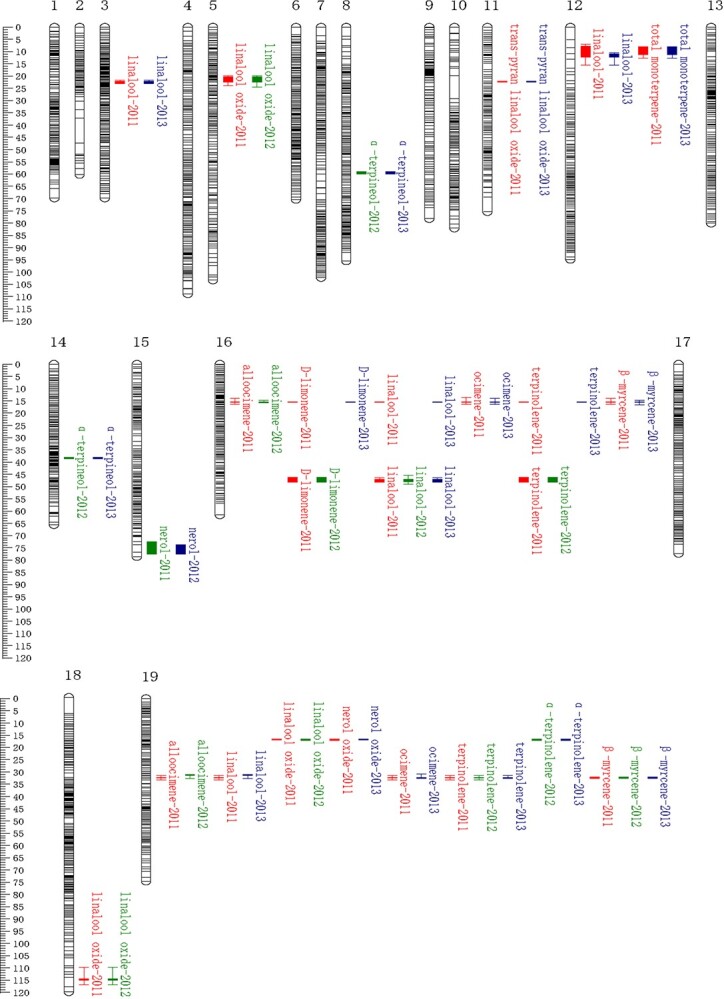
The distribution of QTLs for monoterpene content on the integrated genetic linkage map. Map distances (on the left) are given in cM (Kosambi function). The QTLs of monoterpene compounds named and the years in which they were detected are also labeled in the figure. The confidence intervals of different monoterpene QTLs at LOD-1 and LOD-2 were represented with colored boxes and lines (always on the right of the chromosome).

A total of 11 QTLs were detected for 10 of the continuous monoterpene traits stable in at least two years (Fig. 3; Fig.S7, Table S5, see online supplementary material), with a LOD threshold of 3.5. These stable QTLs accounted for 14.2–45% of the phenotypic variation in the monoterpene compounds (Table S5, see online supplementary material) and were mainly located on LGs 12, 16, and 19 (Fig. 3). One of the QTLs was known from previous studies. The known QTL for linalool variation was identified on LG12, which explained 21.6% of the variance in 2011 and 14.7% in 2013 [19]. Interestingly, the QTL on LG12 is also significantly linked with the variation in total monoterpene content, as it explained 42.6% and 18.3% of the variance in total monoterpenes in 2011 and 2013, respectively. The other 10 QTLs were novel. A stable QTL was detected on LG19 for linalool variation in berries harvested in 2011 and 2013, which explained 45% of the 2013 variance in linalool. Another QTL for trans-pyran linalool oxide was identified on LG11, which explained 22.4% of the variance in 2011 and 42.2% in 2013 (Fig. 3; Fig.S7, Table S5, see online supplementary material).

The QTLs for the three discretely segregated monoterpene traits, namely the nerol, linalool 3,7-oxide, and rose oxide content (Fig. S3, see online supplementary material), were detected using the Kruskal–Wallis test with a significance level of 0.005 to take multiple testing into account (Table S6, see online supplementary material). None QTL was stable for at least two years detected using the Kruskal–Wallis test with a significance level of 0.005. Therefore, we further made a classical QTL detection considering only the offspring with detectable levels of these compounds. This analysis revealed one QTL at LG15 related to nerol content in two consecutive years (Table S6, see online supplementary material).

In total, 12 stable QTLs passed either the LOD threshold of 3.5 for a given monoterpene compound in at least two years (Fig. S7, see online supplementary material). To identify candidate genes potentially involved in monoterpene accumulation, we identified the 2075 genes (Table S7, see online supplementary material) located within these stable QTLs (Fig. 1).

### Integration of RNA-seq data with the stable QTL map associates the *VvbZIP61* gene with monoterpene accumulation

RNA expression in mature berries of 34 grape genotypes with distinct aroma profiles was analysed by RNA-seq (Table S8 and Fig. S8, see online supplementary material). A total of 102 samples (34 genotypes each with three biological replicates) were sequenced producing 2.18 billion clean reads in total with an average of 64.2 million reads per accession (Table S8, see online supplementary material). These reads were mapped to the grape reference genome PN40024 12X.v2 [49] with an average mapping rate of 72.5% (Table S8, see online supplementary material). The mapped reads were then used to calculate gene expression levels with Cufflinks software. The number of expressed genes ranged from 10 056 in cv. Ryoho to 10 102 in cv. Jingxiu. Meanwhile, we quantified the monoterpene content of the berries of the 34 genotypes. Total monoterpene content ranged from 2.53 to 1408.61 μg/kg berry fresh weight (Fig. S8a, see online supplementary material). The most abundant monoterpenes were linalool oxide, linalool, and D-limonene, which overall accounted for 29%, 24%, and 22% of the total monoterpene, respectively (Fig. S8b, see online supplementary material). We investigated whether there was any correlation between levels of mRNAs and the total monoterpene content by calculating the Spearman coefficient. Only relationships with total monoterpene content were tested in this correlation analysis because the most dominant monoterpene compounds were known to be correlated with each other (Fig. S8c, see online supplementary material). The expression of 63 genes was significantly correlated with the total monoterpene content (Fig. 1; Table S9, see online supplementary material, |r| > 0.66, adjusted *P* < 0.05).

To select pertinent candidate genes for further study, we focused on genes that were both significantly correlated with the total monoterpene content in the 34 genotypes (Table S9, see online supplementary material) and located within the confidence intervals of the 12 stable QTLs (Table S7, see online supplementary material). Five candidate genes fulfilled these criteria (Fig. 1; Table S10, see online supplementary material). One gene encoded a transcription factor, while the remaining four genes were classified as structural genes. For the remaining work, we chose to focus on the transcription factor.

**Figure 4:**
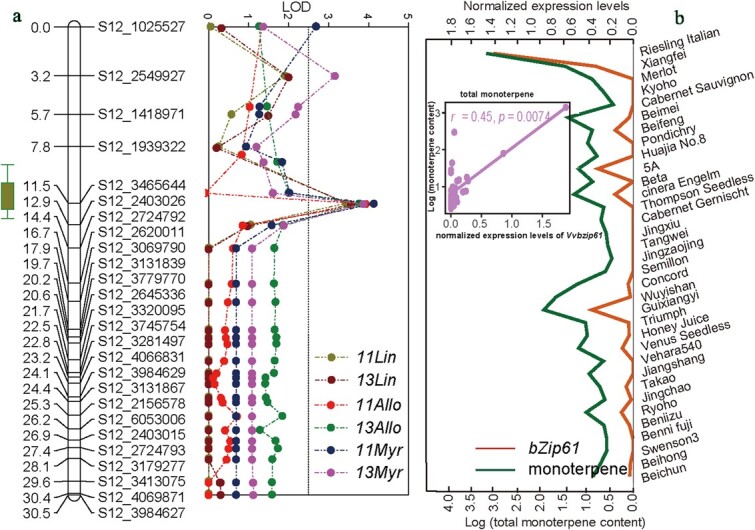
The QTL on linkage group 12 and the association between *VvbZIP61* and monoterpene content in mature berries. **a** QTL for linalool and total monoterpene content on linkage group 12 of the Beifeng (female) × 3–34 (male) map obtained with phenotypic data from the years 2011 and 2013. **b** The Spearman correlation of *VvbZIP61* mRNA expression with monoterpene accumulation of mature fruit from 34 cultivars. *VvbZIP61* mRNA expression was determined by RNA-seq in the mature fruit from 34 cultivars. Monoterpene contents are expressed in logarithmic form.

The *VIT_212s0028g02590* gene encodes *VvbZIP61*, belonging to the basic-leucine zipper (bZIP) transcription factor family known to be involved in morphogenesis and organ development in grapevine [51]. Notably, *VvbZIP61* expression showed the most significant positive correlation with the total monoterpene content (r = 0.45, Fig. 4). Considering individual monoterpene levels separately in the 34 genotypes, Spearman correlation analysis indicated that the expression of *VvbZIP61* was significantly correlated with levels of two different monoterpenes (namely ocimene and linalool oxide), with correlation coefficients (r) ranging from 0.35 to 0.37 (*P* < 0.05) (Fig. S9, see online supplementary material). The associations between *VvbZIP61* expression and monoterpene accumulation were verified by using RT-PCR to quantify *VvbZIP61* mRNA in seven genotypes with distinct monoterpene profiles (Table S11, see online supplementary material). The mRNA levels of *VvbZIP61* were 8 to 68 folds higher in muscat-aroma genotypes (‘Riesling Italian’, ‘Xiangfei’, ‘360’ (F_1_ progeny), ‘3–34’) than in neutral genotypes (‘Beifeng’, ‘Jingzaojing’, ‘243’ (F_1_ progeny)) (Fig. S10, see online supplementary material). The positive correlation between *VvbZIP61* expression and monoterpene content in grape berries indicates that *VvbZIP61* may positively regulate the accumulation of monoterpenes. We considered *VvbZIP61* located on LG12 to be the most pertinent gene for more detailed functional characterization.

### Overexpression of *VvbZIP61* in grape callus increased monoterpene content

**Figure 5:**
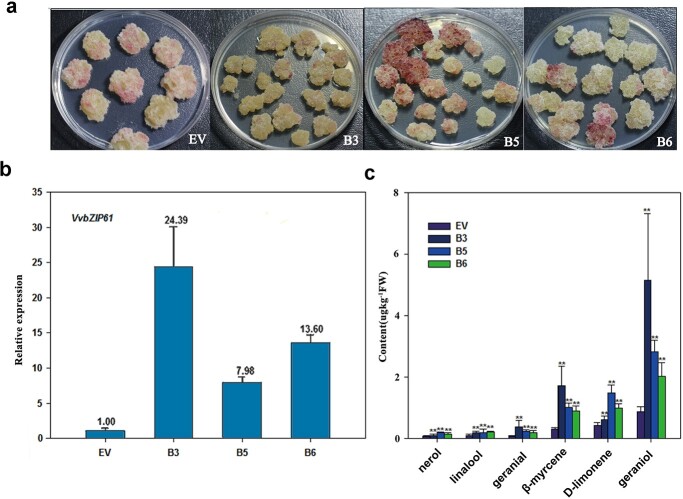
The expression of *VvbZIP61* in transgenic callus and increased monoterpene content. **a***V. amurensis*petiole callus transformed with the empty vector (without the *VvbZIP61* sequence, EV) and different lines of 35S-*VvbZIP61* transgenic callus (B3, B5, and B6). **b** Expression of *VvbZIP61* in EV and transgenic B3, B5, and B6 callus. The y-axis represents the fold difference in gene expression compared with EV (expression standardized as 1). Error bars indicate the standard deviation (SD) from six biological replicates. **c** Nerol, linalool, geranial, β-myrcene, D-limonene, and geraniol content (μg kg^−1^ berry fresh weight) in EV callus and the transgenic B3, B5, and B6 callus. The error bars for EV show the SD of nine biological replicates. The error bars for B3 and B5 show the SD of six biological replicates. The error bars for B6 show the SD of seven biological replicates. The mark ** represents the significant difference *P* < 0.01 according to the *t* test.

To test the function of *VvbZIP61* in monoterpene accumulation, we aimed to generate a transgenic callus overexpressing *VvbZIP61*. Our preliminary attempt showed that it was difficult to induce transgenic callus from ‘Beifeng’ and ‘3–34’, the two parents of the F_1_ population. As an alternative, we transformed petioles of *Vitis amurensis*, which has a neutral aroma background and obtained transgenic callus overexpressing *VvbZIP61* (Fig. 5a). Expression of *VvbZIP61*, measured by qRT-PCR, was compared between control callus transformed with empty vector (EV) and callus transformed with a constitutive expression plasmid containing *VvbZIP61* driven by the 35S promoter. The expression of *VvbZIP61* was 24, 8, and 14-fold higher in the transgenic lines B3, B5, and B6, respectively, than in EV controls (Fig. 5b).

According to GC–MS results the *VvbZIP61* over-expressing callus (*V. amurensis*) contained significantly more monoterpenes than EV (Fig. 5c). The different transgenic lines B3, B5, and B6 contained significantly different amounts of nerol, linalool, geranial, geraniol, β-myrcene, and D-limonene than EV. The highest contents of these six monoterpenes were observed in B3, which was in accordance with the observed expression level of *VvbZIP61* in this line. There was at least twice as much geraniol, geranial, β-myrcene, and linalool in each of the different transgenic calluses as in EV.

## Discussion

### Genetic maps based on inter-specific germplasm

In this study, we applied the GBS method to an F_1_ grapevine population and their parents and generated a genetic linkage map, with a relatively moderate number of SNPs compared to other maps based on SNP markers [35–41, 52–78]. The map constructed in this study was notably from an interspecific cross population (*V. vinifera* × *V. thunbergii*), where *V. thunbergii* is a wild species native to China bearing berries with neutral flavors [6]. The information on these polymorphisms may be of practical use when making inter-species F_1_ population hybrids with the same background to broaden genetic diversity in future grape breeding and may generally strengthen our understanding of genetic determinisms in grapevine.

### The variability extent of monoterpene content

In the years 2011, 2012, and 2013, the raw monoterpene compound contents in the current population (Beifeng × 3–34) ranged from 0 to 1045 μg/kg of berry for linalool, 0 to 173 μg/kg of berry for nerol, and 0 to 64 μg/kg of berry for geraniol. The content ranges for other monoterpene compounds ranged from 0 to 173, 104, 52, 102, 99, 38, 38, 40, 74, 6, 358, 144, 170 and 138 μg/kg of berry for β-myrcene, terpinolene, linalool oxide, ocimene, hotrienol, alloocimene, geranial, trans-pyran linalool oxide, nerol oxide, 4-terpineol, α-terpineol, D-limonene, linalool 3,7-oxide, and rose oxide. In comparison to previously studied populations, it is interesting to note that the TP2687–85 (Olivette × Ribol) × Muscat of Hamburg [18] and Moscato Bianco × *V. riparia* [19] and Italia × Big Perlon [19] population exhibited higher contents of nerol and geraniol. Specifically, nerol ranged from 0 to 802, 400, and 900 μg/kg of berry in these populations, while geraniol ranged from 0 to 856, 900, and 3300 μg/kg of berry, respectively. The linalool range in these populations, except for the Italia × Big Perlon population (range: 0 to 4800 μg/kg of berry), was generally lower than what was observed in our population. In cross 87–1 (*V. vinifera*) × 9–22 (*V. vinifera*) [17], the α-terpineol contents (range: 0 to 425 μg/kg of berry) were comparable to our population (range: 0 to 358 μg/kg of berry). Overall, comparing the raw phenotypic data range allows us to observe differences in monoterpene compound contents between the current population and previously studied populations.

### QTLs for monoterpene accumulation identified

Grape aroma is a polygenic quantitative trait controlled by complex genetic regulatory mechanisms [16, 19]. It is challenging to accurately detect stable aroma QTLs due to the influence of environmental factors, which may significantly influence aroma profiles from year to year [9, 10, 12].

In this study, 12 stable QTLs associated with the amounts of 11 monoterpenes in mature berries were detected in at least two years. One of the QTLs was reported previously for linalool/geraniol located on LG12 [19], which was detected as associated with linalool and total monoterpene in this study. Finding one QTL that has been found by other methods with different germplasms (two mapping populations from crosses of Italia × Big Perlon and Moscato Bianco × *V. riparia*), related to the ratio of linalool/geraniol in Battilana *et al.* [19], is a confirmation that the GBS method for fine mapping with QTL is a valid approach in grapevine.

Four novel QTLs on LGs 16 and 19 were linked with variations in multiple monoterpenes (Fig. 3), which may be attributed to the strong correlations between amounts of individual monoterpenes (Figs S4 andS8c, see online supplementary material) and indicates that a single locus/gene may influence several monoterpene compounds simultaneously. A QTL linked with only one monoterpene, such as the major QTL on LG3 specific to linalool content, which included the gene *HMGR3* (3-hydroxy-3-methylglutaryl-coenzyme A reductase) of Mevalonic acid (MVA). Another QTL only linked with linalool oxide, which included the gene *DXS1* (1-deoxy-D-xylulose-5-phosphate synthase) of methylerythritol phosphate pathway (MEP) (Fig. S11, see online supplementary material) [79]. The *DXS* gene encodes 1-deoxy-D-xylulose-5-phosphate synthase which catalyzes a step of terpene synthesis, and numerous researchers have reported that the *DXS* gene plays a big part in terpene metabolism in grapes [15, 27]. It is possible that the ‘3–34’ parent carries a homozygous genotype for *DXS1*, which may explain why this QTL on LG5 is not associated with linalool, nerol, and geraniol in this population.

Previous studies of monoterpene-associated QTLs mainly focused on linalool, nerol, and geraniol [18, 19], but the present study mapped QTLs for about 17 individual monoterpenes plus the total monoterpene content, providing a more comprehensive view of the genetic regulation of monoterpene biosynthesis.

### From candidate gene through function to flavor?

The 12 stable QTLs detected, including the one known QTL, is a good starting point for functional characterization. We identified five candidate genes by integrating QTL and RNA-seq based correlation analysis. Notably, three of these genes are located within the confidence intervals of novel QTL on LG16, suggesting their potential role in regulating the trait. Further, the presence of the *VvbZIP61* gene on LG12 within the known QTL indicates that this gene may also be important for controlling the trait. Our preliminary experiments on petiole callus suggest that *VvbZIP61* can upregulate monoterpene synthesis, but levels remain below those required for an effect on organoleptic properties. For example, odor threshold values are 3000 μg/kg for nerol 6 μg/kg for linalool, 40 μg/kg for geraniol, 32 μg/kg for geranial, 36 μg/kg for β-myrcene, and 10 ug/kg for limonene [80], which are all higher than the corresponding concentrations found in transgenic callus. While it is probable that the metabolic capacity of *V. amurensis* petiole callus is below that of mature *V. vinifera* berries, the demonstration of *VvbZIP61* function in determining berry aroma is a priority.

Interestingly, the overexpression of *VvbZIP61* in *V. amurensis* callus mainly affected the geraniol content and had a smaller impact on linalool content. This discrepancy in the effect of *VvbZIP61* overexpression on linalool and geraniol contents may be due to several factors, including the complexity of monoterpene biosynthesis in grapevine and the interconnectivity among different branches of the pathway and the different backgrounds of transformation materials, leading to differences in metabolic end products.

It has been reported that the basic leucine zipper family of transcription factors can regulate growth, development, and stress responses [51]. The gene *VvbZIP61* is mainly expressed in pollen and may be involved in flower development and responses to drought and heat [51]. Monoterpenes accumulation is drought-responsive [51] during berry ripening and late-flower developmental stages. The *VvbZIP61* gene is certainly an intriguing candidate as a regulatory gene that warrants further investigation. The possibility the basic leucine zipper family may be involved in monoterpene accumulation is a new perspective in grape genetics*.*

## Conclusion

Grapevine is a major fruit crop distributed worldwide, but grape breeding is still very time-consuming. To facilitate molecular-assisted breeding and gene identification, a mapping population derived from interspecific hybridization between ‘Beifeng’ and ‘3–34’ was constructed. Eleven novel stable QTLs and one known QTL for monoterpene accumulation were detected and associated with candidate genes. We identified *VvbZIP61* and verified that the transcription factor can promote monoterpene accumulation in *Vitis sp. in vitro*, but this regulatory function and mechanism need further research. These results provide new insights into the mechanisms of monoterpene accumulation and would help enhance breeding strategies by using molecular marker-assisted selection for improving grape aroma in grape cultivars.

## Materials and methods

### Plant materials

Identification of QTL for monoterpenes in grape berries was performed using a segregating F_1_ progeny of parents with contrasting flavor characteristics. ‘Beifeng’ was the maternal parent with low monoterpene content originally selected from a cross between *V. thunbergii* and *V. vinifera*, while ‘3–34’ was the paternal parent with a strong muscat flavor originally selected from interspecific hybridization between ‘Jingxiu’ (*V. vinifera*) and ‘Xiangfei’ (*V. vinifera*).

The parents and 150 F_1_ progeny were planted in the vineyard of the Institute of Botany, Chinese Academy of Sciences, Beijing. In addition, 34 cultivars (Table S8, see online supplementary material), including 29 cultivars selected from 62 genotypes in germplasm resources for their aroma profiles [81], and five cultivars selected from our vineyard, were used for monoterpene content and RNA-seq analysis. All materials were grown in the same vineyard and managed with routine cultivation.

### Analysis of monoterpene compounds

We investigated the monoterpene content in the berries of both parents and progenies in three successive years (2011–2013). In parallel, the monoterpene content of the 34 accessions used for RNA-seq was measured in 2013 (Table S8, see online supplementary material). Three replicates of berries were sampled at maturity; each replicate consisted of 20 berries from single bunches. Berries were sampled at similar stages of maturity, assessed by the plateaued sugar and acid content detected with handheld sugar measuring instrument and acidity meter, and the ripened seed color without visible senescence of the berry tissue. Then samples were placed in liquid nitrogen and stored at −80°C waiting for analysis.

The samples were prepared as described by Liu *et al*. [82]. Headspace solid-phase microextraction (HS-SPME) was performed for samples. Pitted frozen grapes (50 g) were pulverized with 5 g of CaCl_2_ to inhibit enzyme activity. Five grams of the homogenate were placed in a 15-ml capped vial with a 10 μL internal standard of 32.84 mg L^−1^ in the solution of 3-octanol ethanol for quantification. The samples were stirred at 40°C. After 20 min equilibration between the solution and the headspace in a vial, the fiber was then inserted into the headspace for 30 min. Then the fiber was inserted into the injection port with spitless mode, desorption at 250°C for 4 min.

Monoterpenes were quantified using the GC–MS method as described by Yang *et al.* [6] and Liu *et al.* [82], using an Agilent 7890 GC equipped with a DB-17MS capillary column (30 m × 0.25 mm × 0.25 μm; J & W, Folsom, CA), binding with an Agilent 5975 C quadrupole mass spectrometer (Agilent, Santa Clara, CA, USA). Monoterpene compounds were searched from the data system library (NIST2008) and published spectra (Mass Spectrometry Data Centre 1974) and supported by the retention index data and retention indices provided in the literature (NIST Chemistry WebBook 2005). The monoterpene compound content was quantified as 3-octanol equivalents. The monoterpene content of the *VvbZIP61* transgenic callus was also detected using this method.

### DNA extraction

F_1_ individuals and the parents’ leaves were sampled in late spring. Then those leaves were placed in liquid nitrogen and frozen at −80°C in the freezer. Leaf samples, approximately 0.5 g, were ground and extracted the DNA. DNA concentration was evaluated by spectrofluorimetry in a Tecan Genios microplate reader with Hoechst 33.258 (Thermofisher).

### SNP identification and genotyping

The Cornell University Genomics Core Laboratory prepared genomic samples with the *ApeKI* restriction enzyme for digestion to construct a library and sequenced them using the IlluminaHiSeq®2000 platform. The *V. vinifera* PN40024 12X.v2 [49] genome sequencing downloaded from the JGI Phytozome website (http://www.phytozome.org/) was used as the reference genome. The TASSEL 3.0 GBS pipeline for alignment and Burrows-Wheeler Aligner (BWA-MEM) with default parameters were used to analyse the raw sequencing data of the 150 F_1_ individuals and the parents (‘Beifeng’ and ‘3–34’). The main steps were discarding reads with low quality, identifying original reads by their barcodes, clustering reads, aligning reads with each other, then scoring SNPs. The SNPs of the two parents (each with three wells and three barcodes) were accessed using a Perl script. After comparing the SNP markers with the genotypes of the parents, all SNPs were classified according to three segregation patterns, lm × ll, nn × np, or hk × hk. Loci were filtered according to two criteria. First, the chi-square test was utilized to evaluate marker segregation against the expected segregation ratio, with the threshold *P*-value set to 0.05. Second, to avoid potential bias due to missing data, any loci with more than 20% missing data were filtered out.

### Map construction and QTL analysis

Linkage maps were constructed using Join Map version 4.0 [50]. The cross-pollination model was utilized after importing the data. Logarithms of odds (LOD) score thresholds ≥9 were used to group markers. The ‘Create Maternal and Paternal Node’ function was applied to create maternal and paternal data sets in the JoinMap. The regression mapping algorithm results in JoinMap was used to construct the map. The ‘Join’ function was then used to combine parental maps. Kosambi’s mapping function [83] was used to calculate JoinMap distances. The distances were shown in centiMorgans (cM). Linkage groups (LGs) were visualized graphically with MapChart 2.2 [84].

QTLs were sought for the quantities of 17 individual monoterpene compounds found in berries harvested in three years and analyzed using Map QTL v5.0 software [80]. Because there was continuous and discrete variation in the contents of different monoterpene compounds (Figs S2 andS3, see online supplementary material), we used different QTL identification methods for each type of variation. Interval mapping and multiple QTL model computations were used to detect the loci of the 14 monoterpenes with a continuous variation. The threshold LOD value was set to 3.5 for all quantitative traits to identify putative QTL [81, 82]. A significant LOD threshold was calculated by a 1000 permutation test at the 95% confidence level (Table S4, see online supplementary material). The Kruskal–Wallis (KW) test was initially performed on the three monoterpene compounds, which exhibited discrete variation (Fig. S3, see online supplementary material). Subsequently, interval mapping and multiple QTL model computations were conducted, utilizing only the offspring with detectable levels. The reliable QTLs were identified based on samples from two or three years or for previously published loci based on samples from one year.

### Mapping of the nerol, linalool 3,7-oxide, and rose oxide qualitative traits

The presence and absence of nerol, linalool 3,7-oxide, and rose oxide in the F1 progeny were segregated in 1:1, 3:1, and 3:1 ratios, respectively (Table S1, see online supplementary material). These monoterpenes were thus treated as qualitative trait loci. The association between markers and the phenotype of three monoterpenes was tested by the non-parametric Kruskal–Wallis test; that is, by single marker regression.

### RNA extraction, library construction, and RNA-sequencing

For the RNA-seq analysis of the 34 accessions (Table S8, see online supplementary material), three clusters of mature berries were chosen at random from three vines for each accession. Thirty berries were sampled from various positions within each cluster, constituting one replicate. This sampling procedure was repeated three times, resulting in three biological replicates for each variety included in the study. The samples were frozen at −80°C and RNA was extracted. The purified total RNA was processed with the NEBNext Ultra RNA Library Prep kit (Cat#E7530, New England Biolabs Corporation, Ipswich, USA) to build cDNA libraries. Library sequencing was carried out by Jingneng Corporation (Shanghai, China).

### RNA-seq data processing, analysis, and selection

Raw reads were first cleaned and quality-controlled using Trimmomatic (v0.36) [85] with the parameter of LEADING:3 TRAILING:3 SLIDINGWINDOW:4:15 MINLEN:30. Three replicate reads for each accession were mapped against the reference genome sequence of grape PN40024 12X.v2 [49] using TopHat (v2.1.1) [86], which allowed no more than a two-nucleotide mismatch. The mRNA levels were calculated with Cufflinks software (v2.2.1) [87]. The RNA-seq data from the 34 cultivars were stored in the National Center for Biotechnology Information (NCBI) Sequence Read Archive (SRA) database (PRJNA565689). Detailed information has been provided in Table S8 (see online supplementary material).

### Spearman correlation analysis between berry mRNA expression and monoterpene content for 34 grapevine accessions

Analysis of covariance between mRNA expression and monoterpene content was done by calculating the Spearman correlation coefficient using the cor. Test function of R [48].

### Statistical analyses

Spearman correlation, using the cor. Test function of R [48], was used to evaluate the correlation coefficient among monoterpene compounds. The statistical test of kurtosis, skewness, and Shapiro–Wilk [47] of R [48] can provide valuable information on the shape and normality of phenotypic distribution. The chi-square test of R [48] was calculated for compound distribution. Cytoscape [88] was used to form the QTL network.

### Quantitative RT-PCR analysis of *VvbZIP61* RNA levels

Total RNA was isolated from ripened berries of 7 genotypes, including muscat-flavored genotypes ‘Riesling Italian’, ‘Xiangfei’, ‘360’ (F_1_ progeny), ‘3–34’ and neutral genotypes ‘Beifeng’, ‘Jingzaojing’, ‘243’ (F_1_ progeny) with three replicates. Total RNA was also isolated from *V. amurensis* callus. Quantitative RT-PCR was then performed. Gene-specific primers VvbZIP61-F1 and VvbZIP61-R1 were designed with the software of Primer 5 (Table S11, see online supplementary material). The 2^−ΔΔCt^ approach was used to calculate genes’ relative expression [89].

### *VvbZIP61* cDNA amplification and sequencing

Total RNA was isolated from 100 mg of leaves of *V. amurensis* with the RNeasy plant kit (TianGen, Beijing, China). Using Superscript III reverse transcriptase (Vazyme, Nanjing, China), first-strand cDNA was synthesized from the total RNA. The *VvbZIP61* cDNA was amplified by PCR with the gene-specific primers VvbZIP61-F2 and VvbZIP61-R2 (Table S11, see online supplementary material). We purified the PCR products from agarose gel, cloned them with a pLB vector, and selected the positive clones. One Shot competent *Escherichia coli* DH5α was used as the host strain for transformation with the plasmid. Positive clones were then sequenced (TsingKe Company, Beijing, China) and analysed using DNAMAN software.

### Overexpression of *VvbZIP61* in *V. amurensis* callus

The full-length cDNA of *VvbZIP61* was ligated into the pSAK277 vector and then transformed into *V. amurensis* embryogenic callus using *Agrobacterium tumefaciens* EHA105. The transformation has been previously described by Zhao *et al.* [90]. The transgenic callus lines were used for PCR and qRT-PCR to confirm the expression of the *VvbZIP61* transgene. The overexpressed positive transgenic calluses were then harvested individually for further analysis.

## Supplementary Material

Web_Material_uhad151

## Data Availability

The data for this manuscript have been deposited in National Center for Biotechnology Information (NCBI) Sequence Read Archive (SRA) database (https://ncbi.nlm.nih.gov/bioproject/PRJNA565689).

## References

[1] Rapp A . Volatile flavour of wine: correlation between instrumental analysis and sensory perception. Die Nahrung. 1998;42:351–63 9881361 10.1002/(sici)1521-3803(199812)42:06<351::aid-food351>3.3.co;2-u

[2] Sáenz-Navajas M-P , Ballester J, Pêcher C et al. Sensory drivers of intrinsic quality of red wines: effect of culture and level of expertise. Food Res Int. 2013;54:1506–18

[3] Lund ST , Bohlmann J. The molecular basis for wine grape quality-a volatile subject. Science. 2006;311:804–5 16469915 10.1126/science.1118962

[4] Hellín P , Manso A, Flores P et al. Evolution of aroma and phenolic compounds during ripening of 'Superior Seedless' grapes. J Agric Food Chem. 2010;58:6334–40 20438135 10.1021/jf100448k

[5] Mateo JJ , Jiménez M. Monoterpenes in grape juice and wines. J Chromatogr A. 2000;881:557–67 10905735 10.1016/s0021-9673(99)01342-4

[6] Yang C , Wang Y, Liang Z et al. Volatiles of grape berries evaluated at the germplasm level by headspace-SPME with GC–MS. Food Chem. 2009;114:1106–14

[7] Fenoll J , Manso A, Hellín P et al. Changes in the aromatic composition of the Vitis vinifera grape Muscat Hamburg during ripening. Food Chem. 2009;114:420–8

[8] Fenoll J , Martínez M-C, Hellín P et al. Changes of free and glycosidically bound monoterpenes and aromatic alcohols in Moscatuel and ruby seedless table grapes during development. OENO One. 2016;46:41

[9] Belancic A , Agosin E, Ibacache A et al. Influence of sun exposure on the aromatic composition of Chilean Muscat grape cultivars Moscatel de Alejandría and Moscatel rosada. Am J Enol Vitic. 1997;48:181–6

[10] Zhang H , Fan P, Liu C et al. Sunlight exclusion from Muscat grape alters volatile profiles during berry development. Food Chem. 2014;164:242–50 24996330 10.1016/j.foodchem.2014.05.012

[11] Dirninger N , Duc DS, Schneider C et al. Wine quality and terroirs: Influence of environmental characteristics on the Gewurztraminer favor profile. Sciences des Aliments. 1998;18:193–209

[12] Reynolds A , Wardle D, Hall J et al. Fruit maturation of four *Vitis vinifera* cultivars in response to vineyard location and basal leaf removal. Am J Enol Vitic. 1995;46:542–58

[13] Reynolds A , Wardle D, Dever M. Vine performance, fruit composition, and wine sensory attributes of Gewürztraminer in response to vineyard location and canopy manipulation. Am J Enol Vitic. 1996;47:77–92

[14] Bueno JE , Peinado R, Moreno J et al. Selection of volatile aroma compounds by statistical and enological criteria for analytical differentiation of musts and wines of two grape varieties. J Food Sci. 2003;68:158–63

[15] Duchene E , Butterlin G, Claudel P et al. A grapevine (*Vitis vinifera* L.) deoxy-D: -xylulose synthase gene colocates with a major quantitative trait loci for terpenol content. Theor Appl Genet. 2009;118:541–52 19002427 10.1007/s00122-008-0919-8

[16] EbangOke JP , Billerbeck GM, Ambid C. Temporal expression of the Lis gene from *Vitis vinifera* L., cv. Muscat de Frontignan. In: *Flavour Research at the Dawn of the Twenty first Century Proceedings of the* 2003 10th Weurman Flavour Research Symposium. Beaune, France, 25–28 June 2002, 321–5

[17] Lin H , Guo Y, Yang X et al. QTL identification and candidate gene identification for monoterpene content in grape (*Vitis vinifera* L.) berries. Vitis-Geilweilerhof. 2020;59:19–28

[18] Doligez A , Audiot E, Baumes R et al. QTLs for Muscat flavor and monoterpenic odorant content in grapevine (*Vitis vinifera* L.). Mol Breed. 2006;18:109–25

[19] Battilana J , Costantini L, Emanuelli F et al. The 1-deoxy-D: -xylulose 5-phosphate synthase gene co-localizes with a major QTL affecting monoterpene content in grapevine. Theor Appl Genet. 2009;118:653–69 19037624 10.1007/s00122-008-0927-8

[20] Lodhi MA , Daly MJ, Ye GN et al. A molecular marker based linkage map of Vitis. Genome. 1995;38:786–94 7672609 10.1139/g95-100

[21] Dalbó MA , Ye GN, Weeden NF et al. A gene controlling sex in grapevines placed on a molecular marker-based genetic map. Genome. 2000;43:333–40 10791822

[22] Doligez A , Bouquet A, Danglot Y et al. Genetic mapping of grapevine (*Vitis vinifera* L.) applied to the detection of QTLs for seedlessness and berry weight. Theor Appl Genet. 2002;105:780–95 12582493 10.1007/s00122-002-0951-z

[23] Welter LJ , Göktürk-Baydar N, Akkurt M et al. Genetic mapping and localization of quantitative trait loci affecting fungal disease resistance and leaf morphology in grapevine (*Vitis vinifera* L). Mol Breed. 2007;20:359–74

[24] Goulão L , Oliveira CM. Molecular characterisation of cultivars of apple (malus × domestica Borkh.) using microsatellite (SSR and ISSR) markers. Euphytica. 2001;122:81–9

[25] Schwander F , Eibach R, Fechter I et al. Rpv10: a new locus from the Asian Vitis gene pool for pyramiding downy mildew resistance loci in grapevine. Theor Appl Genet. 2012;124:163–76 21935694 10.1007/s00122-011-1695-4

[26] Vezzulli S , Malacarne G, Masuero D et al. The Rpv3-3 haplotype and Stilbenoid induction mediate downy mildew resistance in a grapevine interspecific population. Frontiers Plant Science. 2019;10:234 10.3389/fpls.2019.00234 PMC6414455 30894868

[27] Emanuelli F , Battilana J, Costantini L et al. A candidate gene association study on Muscat flavor in grapevine (*Vitis vinifera* L.). BMC Plant Biol. 2010;10:241 21062440 10.1186/1471-2229-10-241 PMC3095323

[28] Battilana J , Emanuelli F, Gambino G et al. Functional effect of grapevine 1-deoxy-D-xylulose 5-phosphate synthase substitution K284N on Muscat flavour formation. J Exp Bot. 2011;62:5497–508 21868399 10.1093/jxb/err231 PMC3223048

[29] Dalla Costa L , Emanuelli F, Trenti M et al. Induction of terpene biosynthesis in berries of microvine transformed with VvDXS1 alleles. Frontiers Plant Science. 2017;8:2244 10.3389/fpls.2017.02244 PMC5776104 29387072

[30] Yu Y , Zhang X, Yuan J et al. Genome survey and high-density genetic map construction provide genomic and genetic resources for the Pacific white shrimp Litopenaeus vannamei. Sci Rep. 2015;5:15612 26503227 10.1038/srep15612 PMC4621519

[31] Elshire RJ , Glaubitz JC, Sun Q et al. A robust, simple genotyping-by-sequencing (GBS) approach for high diversity species. PLoS One. 2011;6:e19379 21573248 10.1371/journal.pone.0019379 PMC3087801

[32] Poland JA , Brown PJ, Sorrells ME et al. Development of high-density genetic maps for barley and wheat using a novel two-enzyme genotyping-by-sequencing approach. PLoS One. 2012;7:e32253 22389690 10.1371/journal.pone.0032253 PMC3289635

[33] Byrne S , Czaban A, Studer B et al. Genome wide allele frequency fingerprints (GWAFFs) of populations via genotyping by sequencing. PLoS One. 2013;8:e57438 23469194 10.1371/journal.pone.0057438 PMC3587605

[34] Sonah H , Bastien M, Iquira E et al. An improved genotyping by sequencing (GBS) approach offering increased versatility and efficiency of SNP discovery and genotyping. PLoS One. 2013;8:e54603 23372741 10.1371/journal.pone.0054603 PMC3553054

[35] Smith HM , Clarke CW, Smith BP et al. Genetic identification of SNP markers linked to a new grape phylloxera resistant locus in *Vitis cinerea* for marker-assisted selection. BMC Plant Biol. 2018;18:360 30563461 10.1186/s12870-018-1590-0 PMC6299647

[36] Fu P , Tian Q, Lai G et al. Cgr1, a ripe rot resistance QTL in Vitis amurensis 'Shuang Hong' grapevine. Hortic Res. 2019;6:67 31231525 10.1038/s41438-019-0148-0 PMC6544659

[37] Tello J , Roux C, Chouiki H et al. A novel high-density grapevine (*Vitis vinifera* L.) integrated linkage map using GBS in a half-diallel population. Theor Appl Genet. 2019;132:2237–52 31049634 10.1007/s00122-019-03351-y

[38] Possamai T , Wiedemann-Merdinoglu S, Merdinoglu D et al. Construction of a high-density genetic map and detection of a major QTL of resistance to powdery mildew (*Erysiphe necator* Sch.) in Caucasian grapes (*Vitis vinifera* L.). BMC Plant Biol. 2021;21:528 34763660 10.1186/s12870-021-03174-4 PMC8582213

[39] Alahakoon D , Fennell A, Helget Z et al. Berry anthocyanin, acid, and volatile trait analyses in a grapevine-interspecific F2 population using an integrated GBS and rhAmpSeq genetic map. Plants (Basel). 2022;11:696 35270166 10.3390/plants11050696 PMC8912348

[40] Lytkin K , Nosulchak V, Agakhanov M et al. Development of a high-density genetic map for Muscadine grape using a mapping population from selfing of the perfect-flowered vine 'Dixie'. Plants (Basel). 2022;11:3231 36501271 10.3390/plants11233231 PMC9738875

[41] Sapkota S , Chen LL, Yang S et al. Construction of a high-density linkage map and QTL detection of downy mildew resistance in *Vitis aestivalis*-derived 'Norton'. Theor Appl Genet. 2019;132:137–47 30341491 10.1007/s00122-018-3203-6

[42] Wang Z , Gerstein M, Snyder M. RNA-Seq: a revolutionary tool for transcriptomics. Nat Rev Genet. 2009;10:57–63 19015660 10.1038/nrg2484 PMC2949280

[43] Fan Y , Wang Q, Kang L et al. Transcriptome-wide characterization of candidate genes for improving the water use efficiency of energy crops grown on semiarid land. J Exp Bot. 2015;66:6415–29 26175351 10.1093/jxb/erv353 PMC4588889

[44] Xiao Y , Ji Q, Gao S et al. Combined transcriptome and metabolite profiling reveals that IiPLR1 plays an important role in lariciresinol accumulation in *Isatis indigotica*. J Exp Bot. 2015;66:6259–71 26163698 10.1093/jxb/erv333 PMC7107596

[45] Yendrek CR , Koester RP, Ainsworth EA. A comparative analysis of transcriptomic, biochemical, and physiological responses to elevated ozone identifies species-specific mechanisms of resilience in legume crops. J Exp Bot. 2015;66:7101–12 26324463 10.1093/jxb/erv404 PMC4765784

[46] Zhang H , Wang H, Yi H et al. Transcriptome profiling of Cucumis melo fruit development and ripening. Hortic Res. 2016;3:16014 27162641 10.1038/hortres.2016.14 PMC4847005

[47] Shapiro SS , Wilk MB. An analysis of variance test for normality. Biometrika. 1965;52:591–611

[48] R Core Team . R: a language and environment for statistical computing. 2011;1:12–21

[49] Canaguier A , Grimplet J, Di Gaspero G et al. A new version of the grapevine reference genome assembly (12X.v2) and of its annotation (VCost.v3). Genomics Data. 2017;14:56–62 28971018 10.1016/j.gdata.2017.09.002 PMC5612791

[50] Ooijen JW. JoinMap® 4, Software for the calculation of genetic linkage maps in experimental populations. 2006.

[51] Liu J , Chen N, Chen F et al. Genome-wide analysis and expression profile of the bZIP transcription factor gene family in grapevine (*Vitis vinifera*). BMC Genomics. 2014;15:281 24725365 10.1186/1471-2164-15-281 PMC4023599

[52] Zou C , Karn A, Reisch B et al. Haplotyping the Vitis collinear core genome with rhAmpSeq improves marker transferability in a diverse genus. Nat Commun. 2020;11:413 31964885 10.1038/s41467-019-14280-1 PMC6972940

[53] Riaz T , Shehzad W, Viari A et al. ecoPrimers: inference of new DNA barcode markers from whole genome sequence analysis. Nucleic Acids Res. 2011;39:e145 21930509 10.1093/nar/gkr732 PMC3241669

[54] Blanc S , Wiedemann-Merdinoglu S, Dumas V et al. A reference genetic map of Muscadinia rotundifolia and identification of Ren5, a new major locus for resistance to grapevine powdery mildew. Theor Appl Genet. 2012;125:1663–75 22865124 10.1007/s00122-012-1942-3

[55] Venuti S , Copetti D, Foria S et al. Historical introgression of the downy mildew resistance gene Rpv12 from the Asian species *Vitis amurensis* into grapevine varieties. PLoS One. 2013;8:e61228 23593440 10.1371/journal.pone.0061228 PMC3625174

[56] Barba P , Cadle-Davidson L, Harriman J et al. Grapevine powdery mildew resistance and susceptibility loci identified on a high-resolution SNP map. Theor Appl Genet. 2014;127:73–84 24072208 10.1007/s00122-013-2202-x

[57] Chen J , Wang N, Fang L-C et al. Construction of a high-density genetic map and QTLs mapping for sugars and acids in grape berries. BMC Plant Biol. 2015;15:28 25644551 10.1186/s12870-015-0428-2 PMC4329212

[58] Houel C , Chatbanyong R, Doligez A et al. Identification of stable QTLs for vegetative and reproductive traits in the microvine (*Vitis vinifera* L.) using the 18 K Infinium chip. BMC Plant Biol. 2015;15:205 26283631 10.1186/s12870-015-0588-0 PMC4539925

[59] Zyprian E , Šimon S, Schwander F et al. Efficiency of single nucleotide polymorphisms to improve a genetic map of complex pedigree grapevines. Vitis-Geilweilerhof. 2015;54:29–32

[60] Teh SL , Fresnedo-Ramírez J, Clark MD et al. Genetic dissection of powdery mildew resistance in interspecific half-sib grapevine families using SNP-based maps. Mol Breed. 2017;37:1 28127252 10.1007/s11032-016-0586-4 PMC5226326

[61] Barba P , Loughner R, Wentworth K et al. A QTL associated with leaf trichome traits has a major influence on the abundance of the predatory mite Typhlodromus pyri in a hybrid grapevine population. Hort Res. 2019;6:6 10.1038/s41438-019-0169-8 PMC6804712 31645947

[62] Royo C , Rodriguez-Lorenzo M, Carbonell-Bejerano P et al. Characterization of deletions causing berry-color variation in Garnacha and Tempranillo. Acta Hortic. 2019;463–70

[63] Crespan M , Migliaro D, Larger S et al. Unraveling the genetic origin of ‘Glera’, ‘Ribolla Gialla’ and other autochthonous grapevine varieties from Friuli Venezia Giulia (northeastern Italy). Sci Rep. 2020;10:7206 32350312 10.1038/s41598-020-64061-w PMC7190720

[64] Duchêne É , Dumas V, Butterlin G et al. Genetic variations of acidity in grape berries are controlled by the interplay between organic acids and potassium. Theor Appl Genet. 2020;133:993–1008 31932953 10.1007/s00122-019-03524-9

[65] Fu P , Wu W, Lai G et al. Identifying *Plasmopara viticola* resistance loci in grapevine (*Vitis amurensis*) via genotyping-by-sequencing-based QTL mapping. Plant Physiol Biochem. 2020;154:75–84 32535323 10.1016/j.plaphy.2020.05.016

[66] Sun L , Li S, Jiang J et al. New quantitative trait locus (QTLs) and candidate genes associated with the grape berry color trait identified based on a high-density genetic map. BMC Plant Biol. 2020;20:302 32605636 10.1186/s12870-020-02517-x PMC7325011

[67] Wang H , Yan A, Sun L et al. Novel stable QTLs identification for berry quality traits based on high-density genetic linkage map construction in table grape. BMC Plant Biol. 2020;20:411 32883214 10.1186/s12870-020-02630-x PMC7470616

[68] Hugalde IP , Paolinelli M, Agüero CB et al. Prioritization of vigor QTL-associated genes for future genome-directed Vitis breeding. Revista de la Facultad de Ciencias Agrarias UNCuyo. 2021;53:27–35

[69] Mamani M , Lopez ME, Correa J et al. Identification of stable quantitative trait loci and candidate genes for sweetness and acidity in tablegrape using a highly saturated single-nucleotide polymorphism-based linkage map. Grape Wine Res. 2021;27:308–24

[70] Negus KL , Chen L-L, Fresnedo-Ramírez J et al. Identification of QTLs for berry acid and tannin in a Vitis aestivalis-derived 'Norton'-based population. Fruit Res. 2021;1:1–11

[71] Su K , Guo Y, Zhong W et al. High-density genetic linkage map construction and white rot resistance quantitative trait loci mapping for genus Vitis based on restriction site-associated DNA sequencing. Phytopathology. 2021;111:659–70 33635092 10.1094/PHYTO-12-19-0480-R

[72] Alahakoon D , Fennell A, Helget Z et al. Berry anthocyanin, acid, and volatile trait analyses in a grapevine-interspecific F2 population using an integrated GBS and rhAmpSeq genetic map. Plan Theory. 2022;11:696 10.3390/plants11050696 PMC8912348 35270166

[73] Rist F , Schwander F, Richter R et al. Relieving the phenotyping bottleneck for grape bunch architecture in grapevine breeding research: implementation of a 3D-based phenotyping approach for quantitative trait locus mapping. Horticulturae. 2022;8:907

[74] Hugalde I , Riaz S, Agüero C et al. Studying growth and vigor as quantitative traits in grapevine populations. In: Maia RT, Campos MA Integrated View of Population Genetics. London: IntechOpen, 2019, 9–24

[75] Guillaumie S , Decroocq S, Ollat N et al. Dissecting the control of shoot development in grapevine: genetics and genomics identify potential regulators. BMC Plant Biol. 2020;20:43 31996141 10.1186/s12870-020-2258-0 PMC6988314

[76] Wang Y , Zhang R, Liang Z et al. Grape-RNA: a database for the collection, evaluation, treatment, and data sharing of grape RNA-Seq datasets. Genes (Basel). 2020;11:315 32188014 10.3390/genes11030315 PMC7140798

[77] Crespan M , Migliaro D, Vezzulli S et al. A major QTL is associated with berry grape texture characteristics. OENO One. 2021;55:183–206

[78] Karn A , Zou C, Brooks S et al. Discovery of the REN11 locus from *Vitis aestivalis* for stable resistance to grapevine powdery mildew in a family segregating for several unstable and tissue-specific quantitative resistance loci. Front Plant Sci. 2021;12:733899 34539723 10.3389/fpls.2021.733899 PMC8448101

[79] Wen YQ , Zhong GY, Gao Y et al. Using the combined analysis of transcripts and metabolites to propose key genes for differential terpene accumulation across two regions. BMC Plant Biol. 2015;15:240 26444528 10.1186/s12870-015-0631-1 PMC4595271

[80] Wu Y , Zhang W, Yu W et al. Study on the volatile composition of table grapes of three aroma types. LWT. 2019;115:108450

[81] Liu X , Fan P, Jiang J et al. Evolution of volatile compounds composition during grape berry development at the germplasm level. Sci Hortic. 2022;293:110669

[82] Liu C , Fan P, He M et al. Inheritance of Muscat berry volatiles in grape interspecific cross population. Euphytica. 2016;208:73–89

[83] Kosambi DD . The estimation of map distances from recombination values. Ann Eugenics. 1943;12:172–5

[84] Voorrips RE . MapChart: software for the graphical presentation of linkage maps and QTLs. J Hered. 2002;93:77–8 12011185 10.1093/jhered/93.1.77

[85] Bolger AM , Lohse M, Usadel B. Trimmomatic: a flexible trimmer for Illumina sequence data. Bioinformatics. 2014;30:2114–20 24695404 10.1093/bioinformatics/btu170 PMC4103590

[86] Kim D , Pertea G, Trapnell C et al. TopHat2: accurate alignment of transcriptomes in the presence of insertions, deletions and gene fusions. Genome Biol. 2013;14:R36 23618408 10.1186/gb-2013-14-4-r36 PMC4053844

[87] Trapnell C , Roberts A, Goff L et al. Differential gene and transcript expression analysis of RNA-seq experiments with TopHat and cufflinks. Nat Protoc. 2012;7:562–78 22383036 10.1038/nprot.2012.016 PMC3334321

[88] Shannon P , Markiel A, Ozier O et al. Cytoscape: a software environment for integrated models of biomolecular interaction networks. Genome Res. 2003;13:2498–504 14597658 10.1101/gr.1239303 PMC403769

[89] Livak KJ , Schmittgen TD. Analysis of relative gene expression data using real-time quantitative PCR and the 2−ΔΔCT method. Methods. 2001;25:402–8 11846609 10.1006/meth.2001.1262

[90] Zhao T , Wang Z, Su L et al. An efficient method for transgenic callus induction from Vitis amurensis petiole. PLoS One. 2017;12:e0179730 28640905 10.1371/journal.pone.0179730 PMC5481001

