# Supplementary material for: Basic leucine zipper gene VvbZIP61 is expressed at a quantitative trait locus for high monoterpene content in grape berries
Source: Hortic Res. 2023 Jul 18;10(9):uhad151. doi: 10.1093/hr/uhad151 (PMC10493639; doi:10.1093/hr/uhad151)
Supplement: Web_Material_uhad151 [file web_material_uhad151.zip › Supplementary figure.docx]

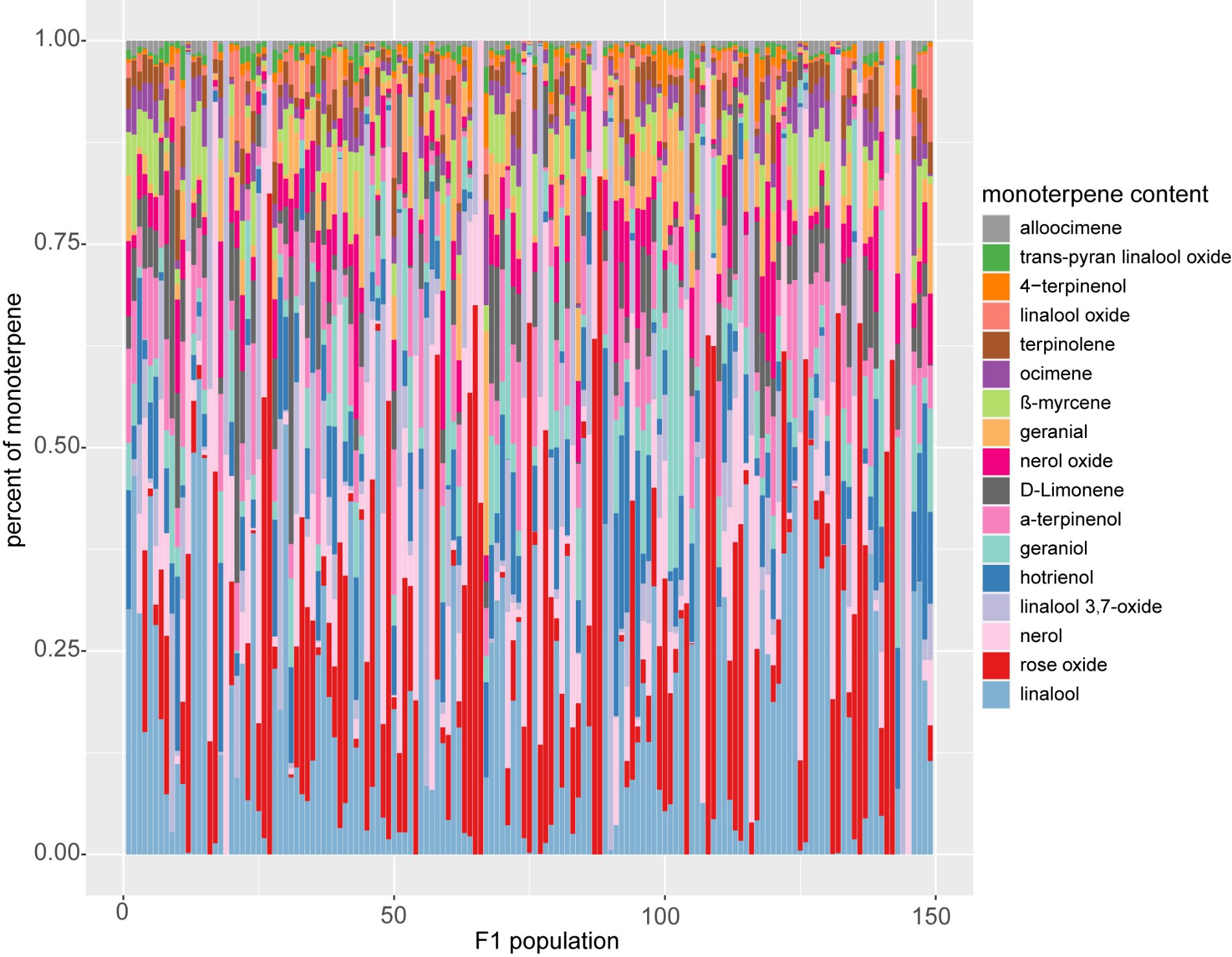


**Figure S1.** Stacked column chart (sorted bottom-up according to mean abundance) showing the percentage monoterpene content in ‘Beifeng × 3-34’ F_1_ population.

**
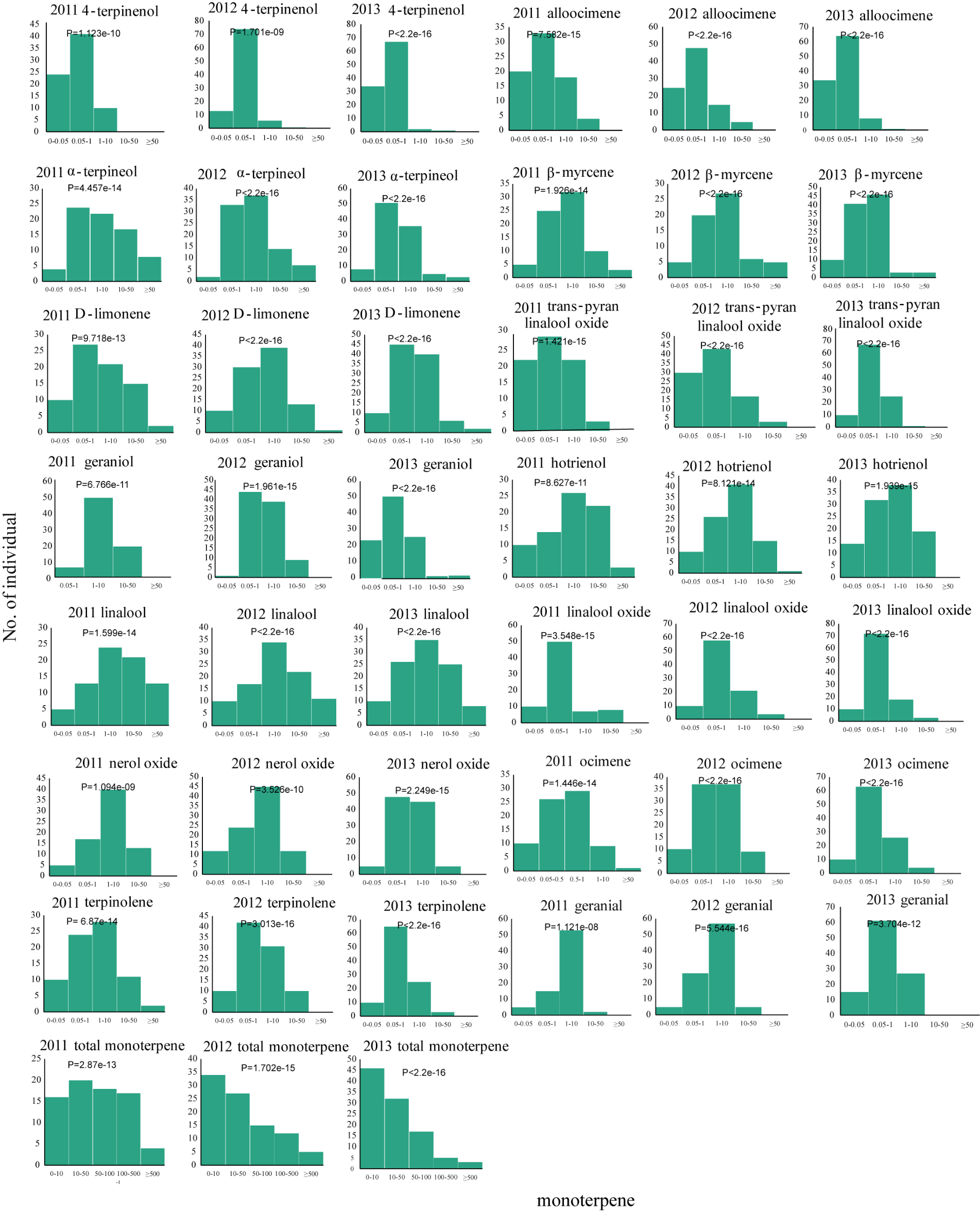
 Figure S2.** Raw distribution of monoterpene compounds (µg/kg berry fresh weight) contributing to the phenotypes of the ‘Beifeng × 3-34’ F_1_ population in 2011, 2012, and 2013. The normal distribution of these compounds was tested using the Shapiro-Wilk test, and the correponding p-values are provided.


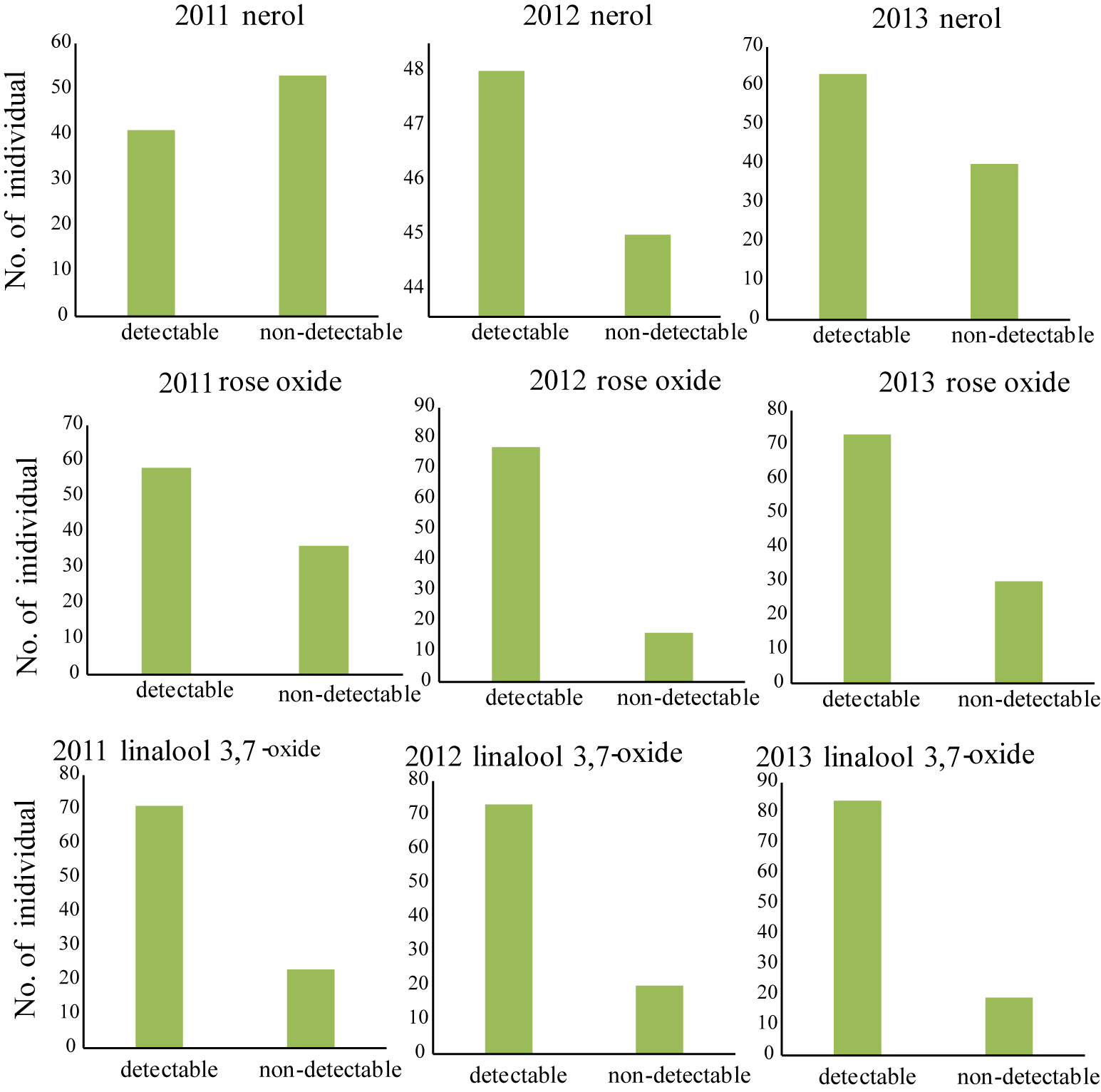


**Figure S3.** Raw distribution of nerol, rose oxide, and linalool 3,7-oxide content (µg/kg berry fresh weight) in the ‘Beifeng × 3-34’ F_1_ population in 2011, 2012, and 2013 population.
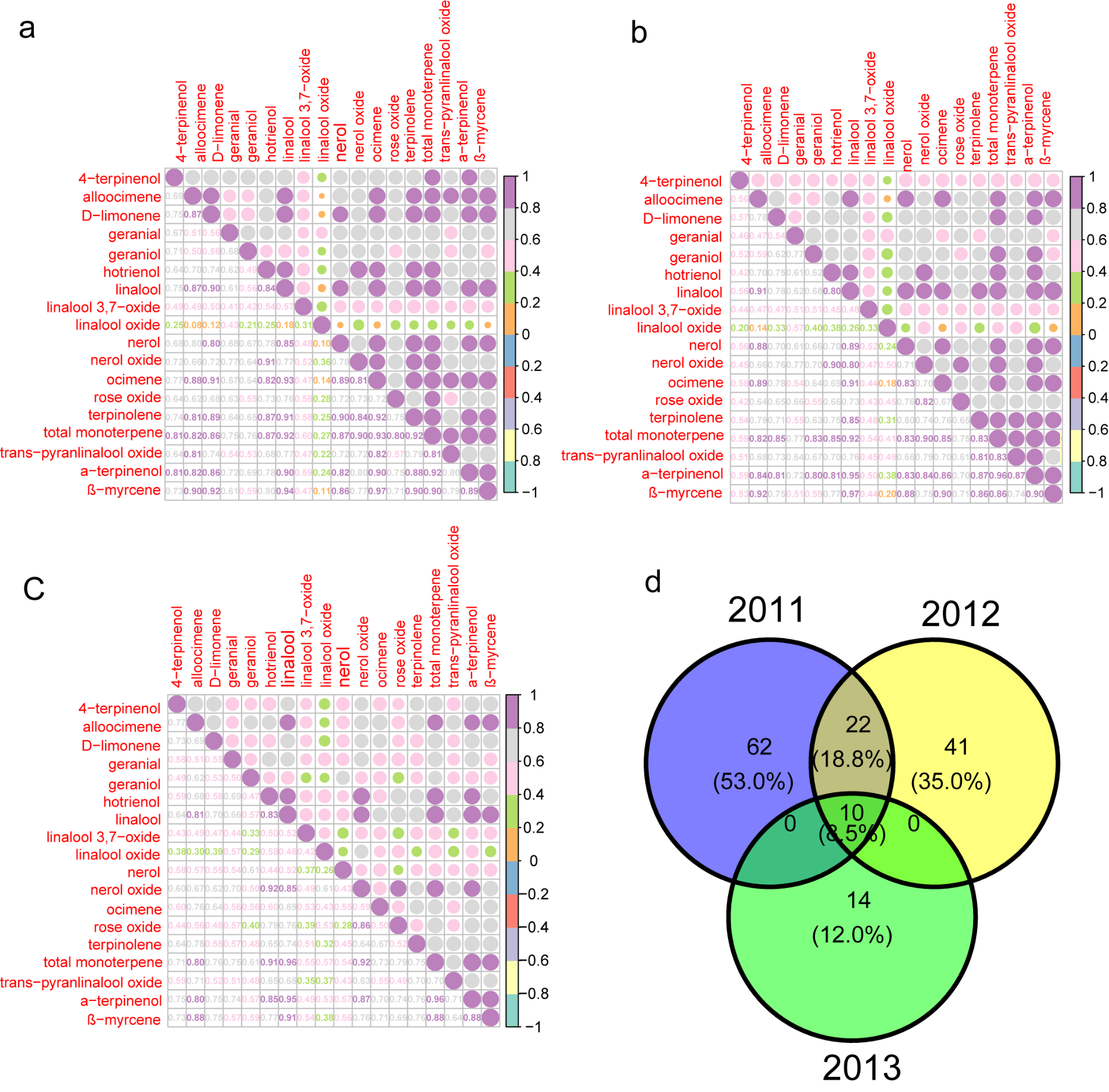
**Figure S4.** The Spearman correlation coefficient among various monoterpene content in the F_1_ population in 2011(a), 2012(b), 2013(c), and those with correlation coefficients greater than 0.8 are counted in 2011, 2012, 2013 (d).


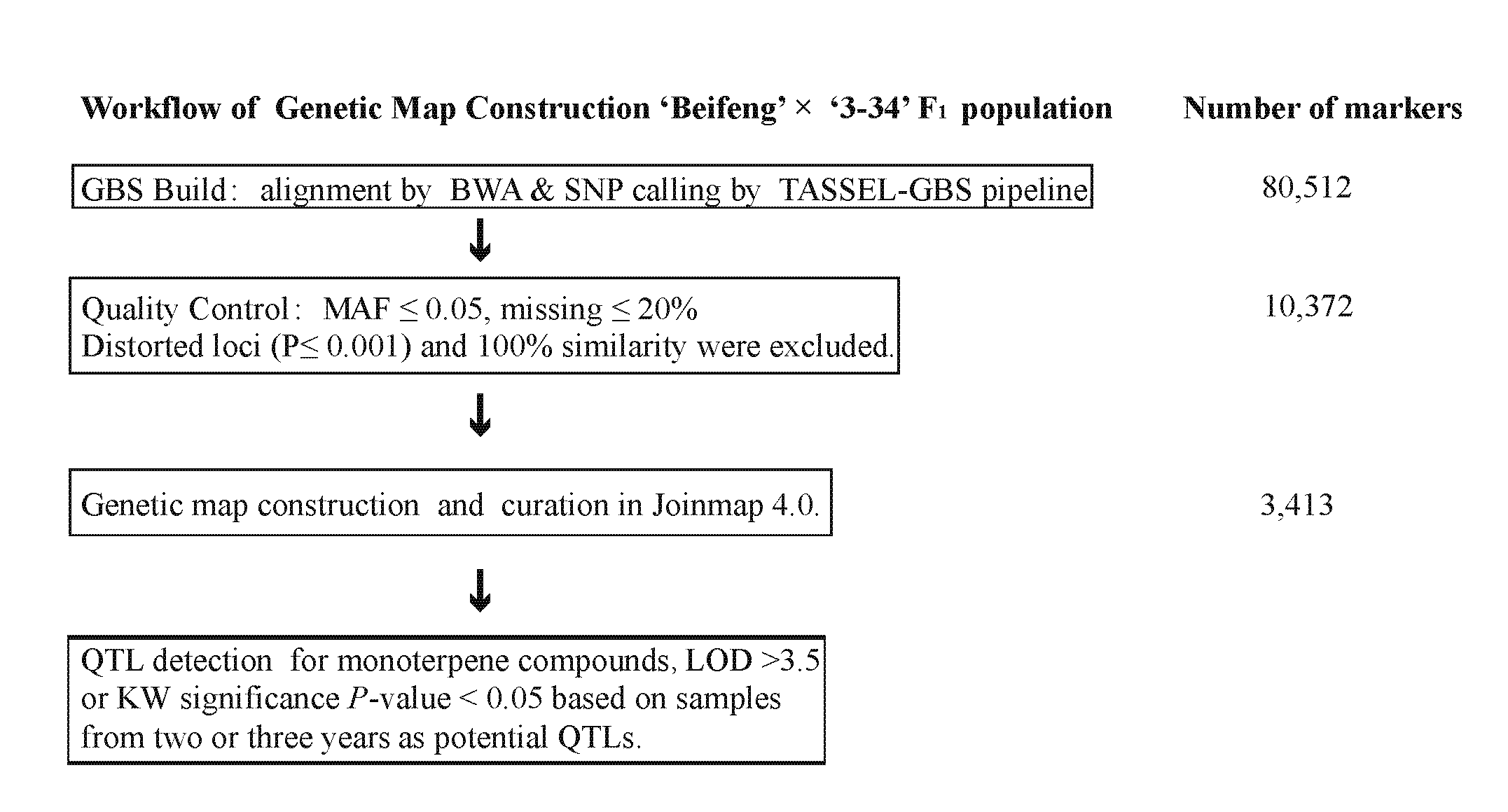


**Figure S5.** Workflow of marker development and genetic map construction in ‘Beifeng × 3-34’ F_1_ population. The left panel shows the four main steps with filtering parameters and the right panel shows the numbers resulting from each step.


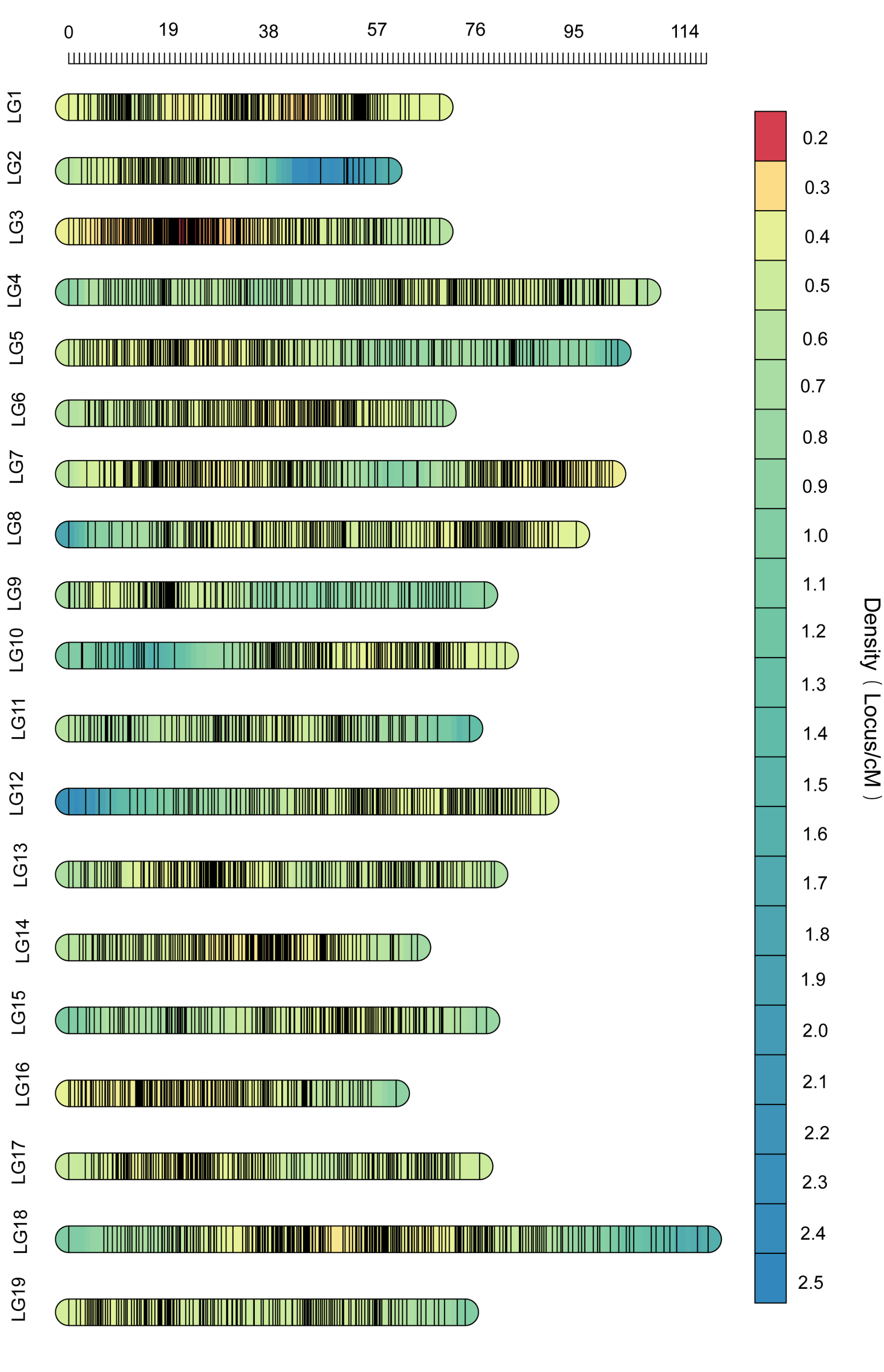


**Figure S6.** The density of integrated linkage map of ‘Beifeng × 3-34’ F_1_ population.


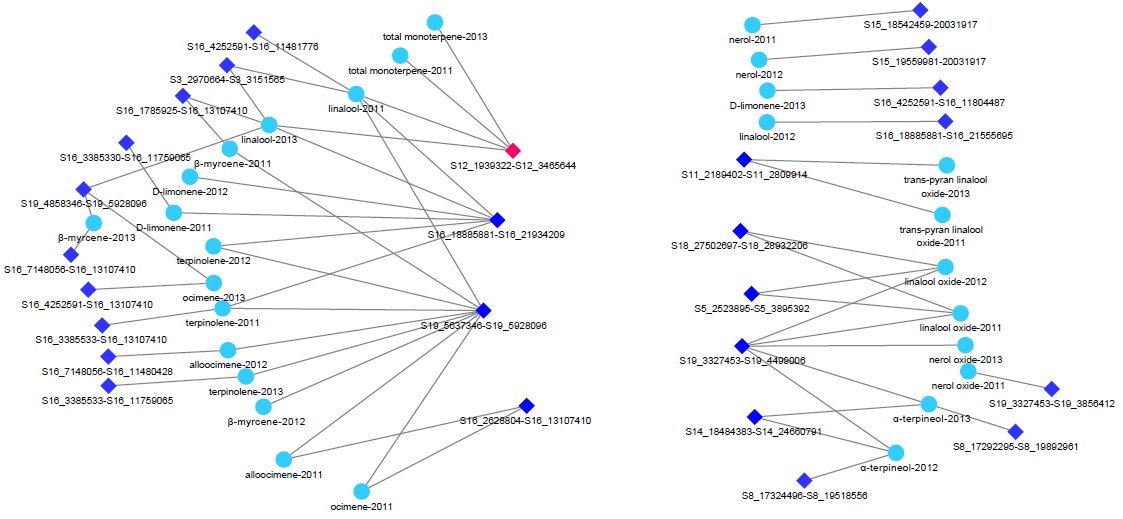


**Figure S7.** The network between QTLs and monoterpene compounds. The diamonds represent QTLs, and the red diamonds represent QTLs located on chromosome 12. The circles represent different individual monoterpenes.


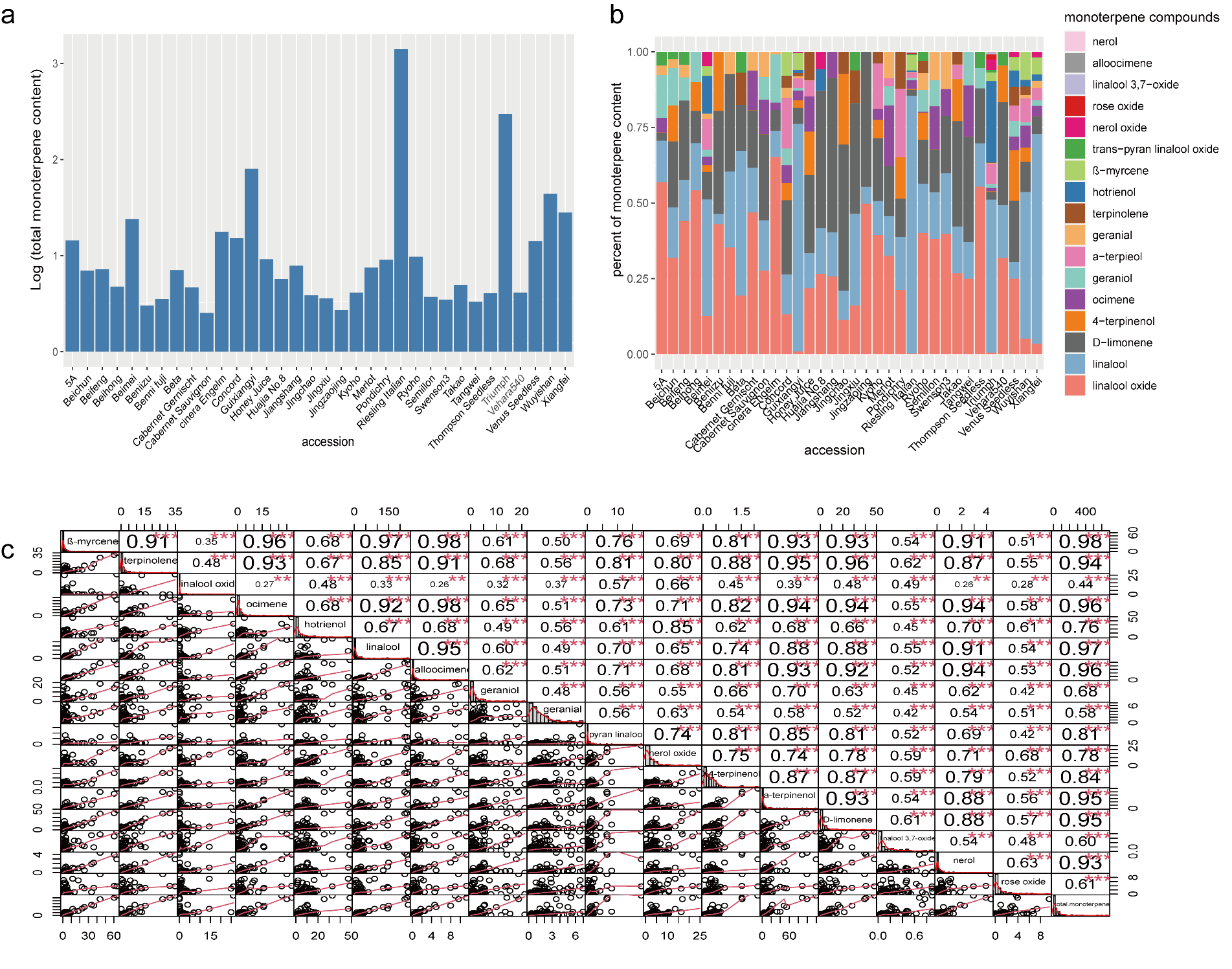


**Figure S8.** The detailed information about monoterpenes content in 34 cultivars. (**a)** The monoterpene content of 34 cultivars. (**b)** Stacked column chart showing percentages of monoterpenes in 34 cultivars. (**c)** The Spearman correlation coefficients between amounts of various monoterpenes in 34 cultivars.


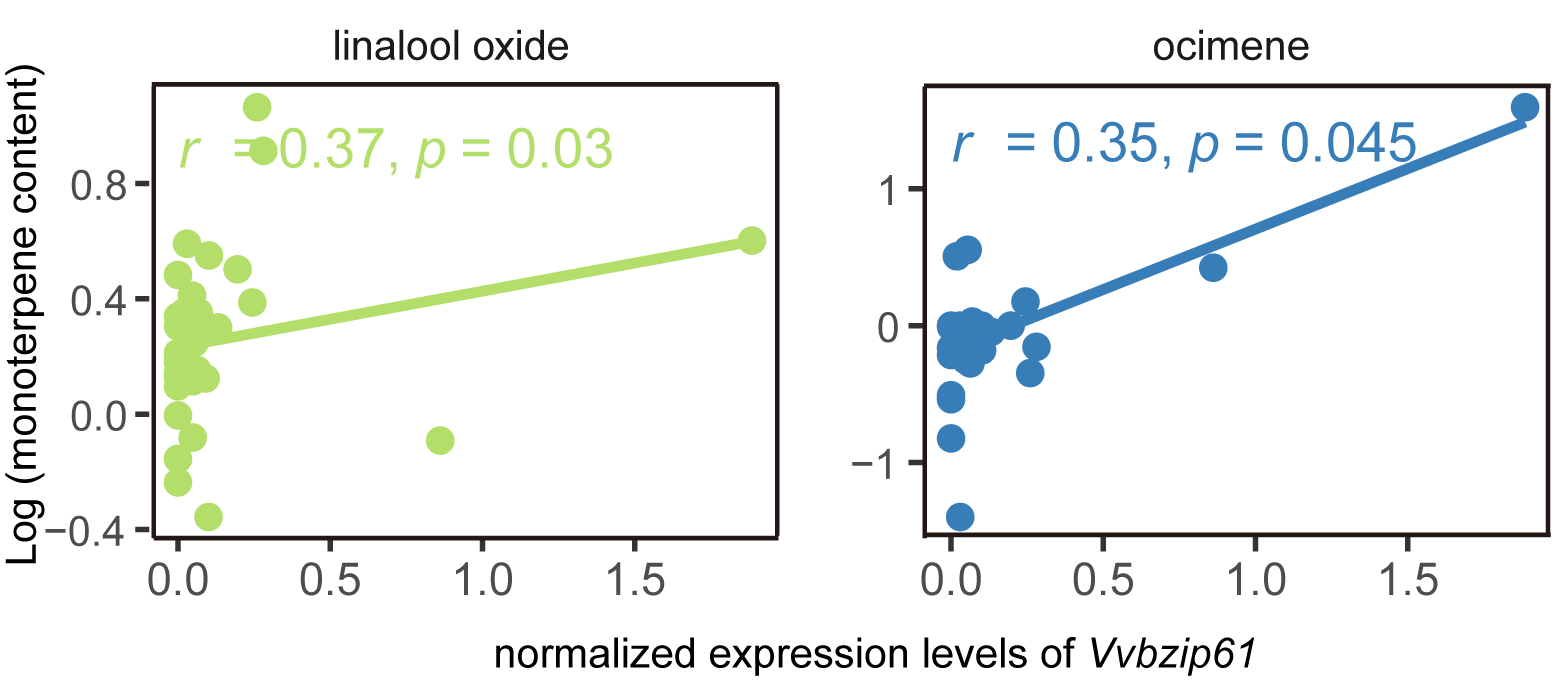


**Figure S9.** The Spearman correlation between amounts of a total monoterpene, 11 different monoterpenes, and the expression of *VvbZIP61*.


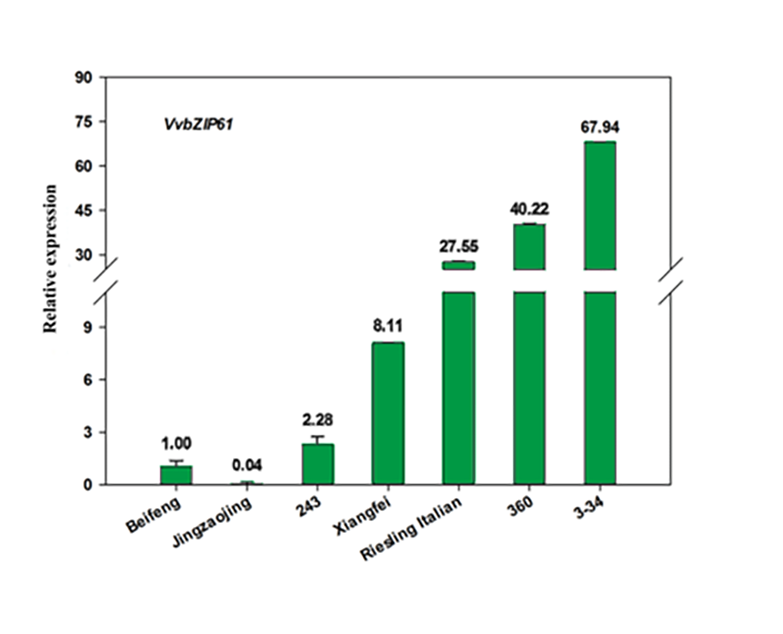


**Figure S10.** qRT-PCR assay results of the relative expression profiles of *VvbZIP61*. The y-axis indicates the fold difference in gene expression compared with Beifeng (expression standardized as 1). The numbers on the x-axis represent the various cultivars: Beifeng (the low monoterpene content parent of the F_1_ population); Jingzaojing (low monoterpene content); F_1_ progeny no. 243 (low monoterpene content); Xiangfei (high monoterpene content); Riesling Italian (high monoterpene content); F_1_ progeny no. 360 (high monoterpene content); and 3-34 (one of the parents in the F_1_ population, high content of monoterpenes). All berries of cultivars were randomly sampled at maturity with three replicates.


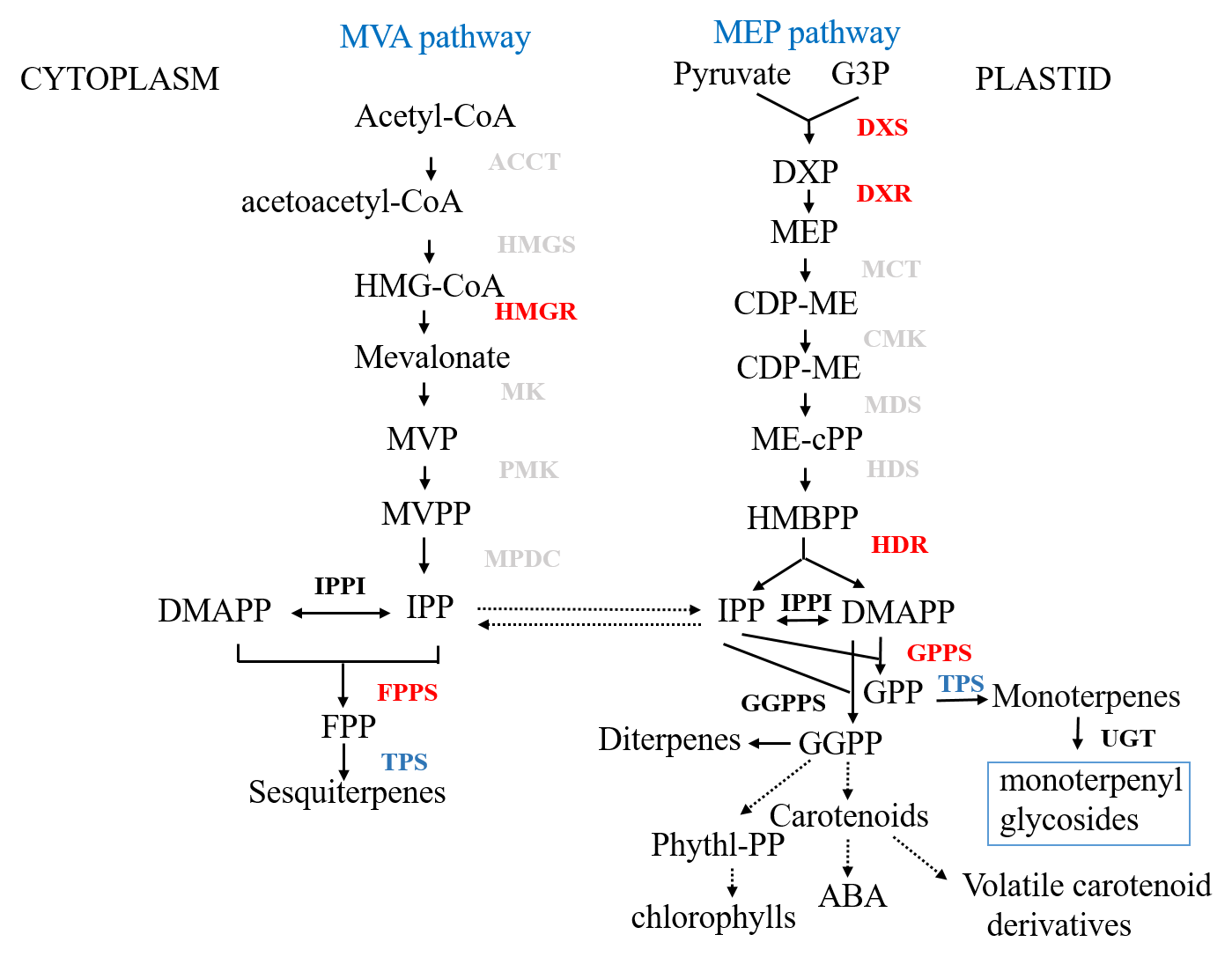


**Figure S11.** Pathway of terpene biosynthesis in grape berries. The MEP pathway is localized in plastids, while the MVA pathway occurs in the cytosol. The red font represents the key synthase genes in the pathway; the blue font represents the genes that perform classes of functions; the black font represents the products in the pathway; the grey font represents the non-key enzyme genes in the pathway.
